# Psychometric performance of the Kannada version of sarcopenia quality of life questionnaire (SarQoL^®^)

**DOI:** 10.1186/s12891-023-06559-8

**Published:** 2023-06-02

**Authors:** Prabal Kumar, Shashikiran Umakanth, N Girish

**Affiliations:** 1 Department of Physiotherapy, Manipal College of Health Professions, Manipal Academy of Higher Education, Manipal, Udupi, Karnataka India; 2 Department of Medicine, Dr. TMA Pai Hospital, Manipal Academy of Higher Education, Udupi, Karnataka India

**Keywords:** Translation, Sarcopenia, SarQol, Questionnaire, Validation

## Abstract

**Background:**

The Sarcopenia Quality of Life (SarQoL®) is a patient reported quality-of-life questionnaire specific to sarcopenia. In the Indian context, its availability is limited to Hindi, Marathi and Bengali vernacular languages.

**Aims:**

This study aimed to translate, cross-culturally adapt the SarQoL® questionnaire into Kannada and investigate its psychometric properties.

**Methods:**

The SarQoL®-English version was translated into Kannada with the developer’s permission and in accordance with their requirements. To validate the discriminative power, internal consistency and floor and ceiling effect of the SarQoL®-Kannada questionnaire were assessed in the first step. In the second step, the construct validity and the test–retest reliability of the SarQoL®-Kannada was determined.

**Result:**

There was no difficulty in the translation process. A total of n = 114 participants (sarcopenic participants n = 45 and n = 69 non-sarcopenic participants) were included. The good discriminative power of the SarQoL®-Kannada questionnaire {quality of life for sarcopenic subjects [56.43 ± 11.32] vs. non-sarcopenic ones [79.38 ± 8.16], p < 0.001}. High internal consistency (Cronbach’s alpha coefficient was 0.904) and no ceiling/ floor effect were reflected. Excellent test–retest reliability (intraclass correlation coefficient was 0.97, 95% CI 0.92–0.98) were found. A good convergent and divergent validity with similar and different domains of WHOQOL-BREF was observed, while EQ-5D-3L had good convergent and weak divergent validity.

**Conclusion:**

The SarQoL®-Kannada questionnaire is valid, consistent and reliable for the measurement of quality of life of sarcopenic participants. SarQoL®-Kannada questionnaire is now available to be used in clinical practice and as a treatment outcome indicator in research.

**Supplementary Information:**

The online version contains supplementary material available at 10.1186/s12891-023-06559-8.

## Introduction

The last ten years of the 20th century saw the introduction of the term “sarcopenia”, which is defined as a decline in muscle mass, muscle strength, and physical performance [1–3]. The prevalence of sarcopenia ranged from 10 to 27% in older adults [4–6]. The European Working Group for Sarcopenia in Older People (EWGSOP) and Asian Working Group for Sarcopenia (AWGS) have come up with the diagnostic criteria for sarcopenia among older adults, which consider muscle mass, muscle strength, and physical performance parameters with slight variations in the cut-off values [1, 2]. A plethora of factors contributes to the development of sarcopenia among older adults, including a sedentary lifestyle, changes in endocrine function (insulin, testosterone, growth hormone, insulin-like growth factor-1, cortisol), loss of neuromuscular function, an imbalance between muscle protein synthesis and breakdown, insufficient dietary protein intake, and genetic factors [7, 8].

A gradual deterioration in the quality of life (QoL) has been evidenced in these individuals. However, much of this research assessing QoL in sarcopenia, till 2015, has been done using generic tools, such as the Short Form 36 questionnaire (SF-36), European Quality of Life-5 Dimensions (EQ-5D), and World Health Organization Quality of Life-BREF (WHOQOL-BREF) which may not be ideal for accurately assessing the impact of sarcopenia on QoL. Consequently, the Sarcopenia Quality of Life (SarQoL®) questionnaire, a disease/ condition-specific measure to assess the impact of sarcopenia on QoL, was developed [9]. The SarQoL® questionnaire is the first multidimensional disease-specific questionnaire designed in the year 2015 for community-dwelling sarcopenic subjects aged 65 years and older. It comprises 22 questions rated on a 3-, 4-, or 5-point Likert scale. Items are categorised into the following sevens domains of dysfunction: physical and mental health, locomotion, body composition, functionality, activities of daily living, leisure activities, and fears. Transcultural adaptation and compatibility studies are required to confirm the instrument’s cultural equivalence and applicability across populations. Generally, determining the applicability or usage of an instrument for clinical use in a clinical setting does not appear to be possible from a simple technical translation from an original version into other vernacular languages [10].

The SarQoL® questionnaire was initially developed and validated in French in 2015 [9, 11] and was later translated and validated into English [12], Dutch [13], Romanian [14, 15], Polish [10], Hungarian [16], Russian [17], Greek [18], Turkish [19] and Ukrainian [20]. With regard to the Indian context, the availability of SarQoL® in the Indian vernacular languages is limited to Hindi, Marathi and Bengali languages. There is a paucity of translation and cross-cultural adaptation of SarQoL® in Kannada language, one of the twenty-two recognised languages in the 8th schedule of the constitution of India spoken in Karnataka state of India. Thus, the objectives of this study were to translate and cross-culturally adapt the SarQoL® questionnaire into Kannada and to determine its psychometric properties.

## Materials and methods

### SarQoL®-Kannada translation

The rights owners of the SarQoL® questionnaire granted permission for translation and cross-cultural adaption [11], and the translation part was sequentially done in five phases as per guidelines [21]: (i) two translations from English to Kannada; (ii) synthesis of the two translations; (iii) backward translations; (iv) compare the backward translations with the original questionnaire by an expert committee and (v) pre-test.

### Phase 1: Initial translations (English to Kannada)

Two bilingual speakers well versed in Kannada and English separately translated the original SarQoL® from English to Kannada. One had a medical background (Intern; Bachelor of Physiotherapy), and the other was a novice in this field (Master of Commerce). The translators were instructed not to do word-by-word translation but instead to retain the meaning of the sentence in the context and provided a written report with comments highlighting difficult words or phrases or uncertainties, as well as the reasons behind specific linguistic choices made. The translators independently translated the questionnaire in a week. The report has been provided in the supplementary material (Supplementary material 1).

### Phase 2: Synthesis

The two translators compared their translations during an offline meeting in a discussion room at the Department of Physiotherapy, Manipal College of Health Professions, which lasted two hours (2 h). The author and both the translators attended the meeting. Each question and item were thoroughly screened for differences. Moreover, the author noted translation discrepancies which reflect potentially ambiguous wordings. The members discussed each difference, reached a consensus, and prepared “Version 1” of the translated questionnaire. A written report was made of this synthesis process, including the actions taken to address and resolve issues that arose. The report has been provided in the supplementary material (Supplementary material 2).

### Phase 3: Backward translations

Two translators (blinded to the original version of the SarQoL®) then independently back translated “Version 1 of Kannada” to the English in forty-five days (45 days). As per the translation guidelines given by the developers, the backward translator’s first language should be English. As it is not possible to get translators with this criterion to any of the Indian vernacular languages, in this study, we contacted English teachers with a Master’s in Education degree, eighteen years (18 years) of experience and no medical background to back translate the questionnaire. These backward translations aim to ensure that Version 1 of Kannada reflects the same item content as the original version.

### Phase 4: Expert committee review

Before conducting the expert committee review meeting, the author identified the discrepancy in the back translated questionnaire with respect to the original questionnaire and prepared a document highlighting the discrepancy. The expert committee meeting was conducted online on the Microsoft Teams video chat (MS Teams) platform considering the feasibility of all the experts. The meeting was attended by one methodologist, three translators (two forward and one backward translator), and one expert. One of the methodologists and a backward translator could not join the meeting because of some unavoidable circumstances. The methodologists led the discussion and clarified the required discrepancies. The meeting went for ninety minutes, and the pre-test version was finalised. The written report of the expert committee review meeting was prepared. The report has been attached as supplementary material (Supplementary material 3).

### Phase 5: Test of the pre-final version

The pre-final version was tested in nineteen (n = 19) older adults, age > 60 years, after obtaining ethics clearance from Institutional Ethics Committee (IEC1: 100/2022) for a larger study which aimed to develop and validate a multi-modal intervention program for sarcopenic older adults. The convenience sampling method was used to recruit the participants. The participants were explained about the questionnaire. If the participant provided oral consent, the author assessed their eligibility by asking about the age and administration of the Strength, Ambulation, Rising from a chair, stair Climbing and history of Falling (SARC-F). The SARC-F questionnaire, with a specificity of 85.7% and positive predictive values of 42.9% [22], was used to screen and categorise older adults with and without Sarcopenia. Total of ten (n = 10) and nine (n = 9) were identified as sarcopenics and non-sarcopenics, respectively. All of them filled in the prefinal version of SarQoL® in the author’s presence, the average time taken was 12 min. After completing the administration, a face-to-face interview was conducted with the individual participants to get feedback about the questionnaire regarding any difficulties/ difficult words or phrases, whether culturally valid or not and also for suggestions. Considering the feedback obtained, the author prepared the document highlighting the difficult words and also included the suggestion of the participant to either make the word simple or replace it and make it more culturally suitable. An email containing the suggestions/ feedback was sent to the experts for their suggestions. After obtaining the responses from the experts, an offline meeting was organised in the conference room at the Department of Physiotherapy, Manipal College of Health Professions. The meeting was attended by two methodologists, two translators and one expert. The members discussed each word and suggestion the participants provided and explored the items to make them more culturally relevant. The necessary modifications were incorporated, and the final version of the Kannada SarQoL® questionnaire was proposed (Supplementary material 4). Further, the SarQoL® -Kannada version questionnaire has been uploaded by the developers on the website (www.sarqol.org). The SarQoL®-Kannada questionnaire is attached as supplementary material 5.

## Validation of SarQoL®-Kannada questionnaire

### Study population

**Fig. 1:**
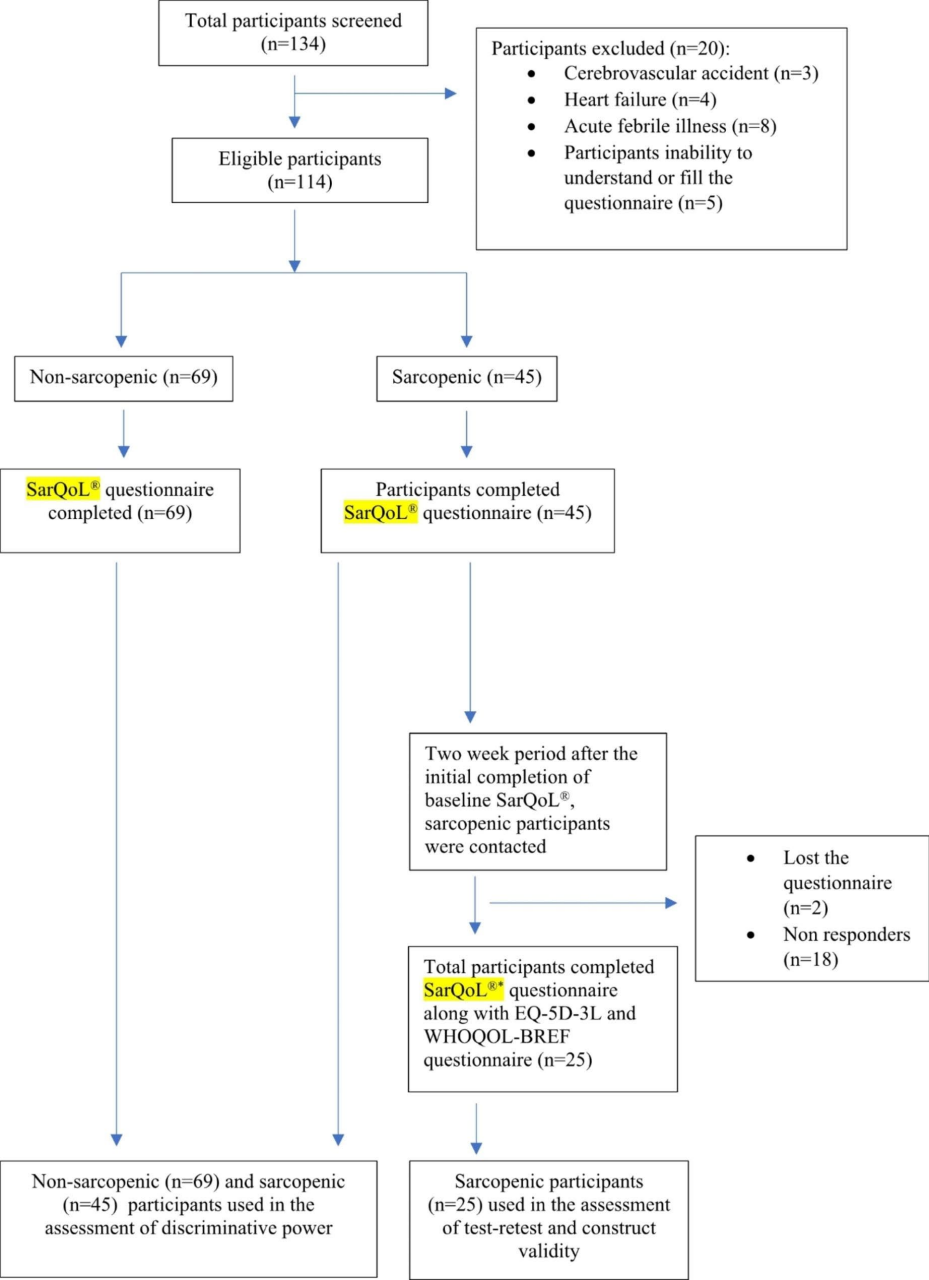
Flowchart of validation study of Kannada version of SarQoL® questionnaire. SarQoL® refers to the baseline SarQoL® used for the “test” and SarQoL®^*^ refers to the SarQoL® used for “retest”

Participants were recruited from the out-patient Department (OPD) of Medicine at Dr. TMA Pai Hospital, Udupi, Karnataka. Inclusion criteria included: (a) either gender, (b) age ≥ 60 years, and (c) a native Kannada speaker who can read. The participants were excluded if: (a) wheelchair bound, (b) pacemaker and any metal implant, (c) history of cerebrovascular accident, heart failure, liver cirrhosis, active tumor, Parkinson’s disease, Alzheimer’s disease, (d) acute febrile illness, and (e) participants inability to understand or fill the questionnaire. The flow of participants is depicted in Fig. 1. The procedure was thoroughly explained to the participants, and their informed consent was obtained. The eligible participants underwent the assessment of their muscle mass, muscle strength and physical performance to categorise them into sarcopenic and non-sarcopenic as per the criteria given by the Asian Working Group for Sarcopenia (AWGS 2019).

### Assessment of sarcopenia

Sarcopenia was defined according to the AWGS 2019 guidelines: low muscle mass and either low physical performance and/or low muscle strength. Skeletal muscle mass was estimated from Omron Karada Scan HBF- 375 Bioelectrical Impedance Analyzer (BIA) measurements and expressed as skeletal muscle mass index (SMI) (SMI = skeletal muscle mass/body mass × 100). The AWGS-2019 cut-off value we used were as follows: <7.0 kg/m^2^ for male participants and < 5.7 kg/m^2^ for female participants. Muscle strength (grip) was assessed with a JAMAR digital handheld dynamometer with the following cut-off values, proposed by AWGS-2019, < 28.0 Kg for male participants and < 18.0 Kg for female participants. Both hands’ grip strength was assessed following the standard criteria, and an average value was obtained. Five time sit-to-stand (5-STS) test was used to evaluate physical performance. Participants were asked to perform STS once. If found to be comfortable, they were asked to repeat it five times as quickly as possible, with the researcher recording the time taken to complete it. Poor physical performance was considered as per the cut-off given by AWGS-2019, with participants taking ≥ 12 s.

### Psychometric properties of the SarQoL®-Kannada questionnaire

Verification of the psychometric properties of the SarQoL®-Kannada questionnaire was conducted according to the original developer’s instructions. The validation of SarQoL questionnaire was assessed as per measurement property according to the COnsensus-based Standards for the selection of health Measurements Instruments (COSMIN) checklist [23]. Specifically, discriminative power, reliability (Internal consistency reliability and test-retest reliability), construct validity (convergent and divergent validity), and floor and ceiling effect were determined.

(A) The discriminative power, internal consistency and floor and ceiling effect of the SarQoL®-Kannada questionnaire was assessed in the first step. All of the analyses described below were performed using IBM Statistical Package for Social Sciences 20. Results were considered statistically significant at p ≤ 0.05.

(1) **Discriminative power:** The null hypothesis was that non-sarcopenic participants have a better quality of life than sarcopenic participants. The SarQoL® score data was found to be normally distributed using the Kolmogorov-Smirnov test; thus, an independent sample T-test was used to assess the difference of overall and domain QoL scores between the sarcopenic participants and the non-sarcopenic participants.

(2) **Internal consistency**: Internal consistency estimates the questionnaire’s homogeneity. To measure internal consistency reliability, we used Cronbach’s alpha coefficient. A coefficient value greater than 0.70 indicates a high level of internal consistency. The impact of each domain on the total score was also considered. The Kolmogorov-Smirnov test tested the normality of quantitative variables. Since scores from the SarQoL®-Kannada questionnaire were normally distributed, the correlation among domains and each domain with the total score of the SarQoL®-Kannada questionnaire were assessed using Pearson’s product-moment correlation (r).

(3) **Floor and ceiling effects**: Floor and ceiling effects were defined when a high percentage of the population had the lowest or the highest score, respectively. Floor and ceiling effects higher than 15% were considered to be significant.

(B) In the second step, the construct validity and the test–retest reliability of the SarQoL®-Kannada was determined. Sarcopenic participants (n = 45) completed the SarQoL® questionnaire, given the SarQoL®*   questionnaire after the interval of 2 weeks as well as the World Health Organization Quality of Life-BREF (WHOQOL-BREF) questionnaire [24] and the EuroQoL 5-dimension 3-levels (EQ-5D-3 L) questionnaire [25]. As the participant visited OPD from different places and got their next appointment after three months, the participants were given the option to send the questionnaire online or the researcher collected the questionnaire from their home and were requested to respect a 2-week interval before completing the SarQoL®* , WHOQOL-BREF, and ED-5D-3 L questionnaires. The researcher gave them a reminder call after the completion of 2-weeks and asked about any change in their health. A total of three reminder calls were made.

(1) **Construct validity**: The construct validity was investigated by measuring using convergent and divergent validity. The correlation between the SarQoL®-Kannada and other questionnaires or domains of questionnaires that were supposed to have similar (convergent validity) or different (divergent validity) dimensions was assessed. Therefore, besides completing the SarQoL®-Kannada, the participants were also asked to complete the WHOQOL-BREF questionnaire composed of four domains (domain 1 physical health, domain 2 psychological, domain 3 social relationship and domain 4 environment). Additionally, participants were also asked to complete the EQ-5D-3 L questionnaire [25], which records the level of self-reported problems according to five dimensions (mobility, self-care, usual activities, pain/discomfort and anxiety/depression), with each dimension having three levels: no problems, some problems and extreme problems.

(2) **Test–retest reliability**: The intraclass correlation coefficient (ICC) was used to test the reliability between the first and second questionnaires overall and individual domain scores of the SarQoL®-Kannada questionnaire. An ICC over 0.7 was considered acceptable reliability [26]. The subjects were enquired about any health change (physical and mental health; e.g., sickness, fall, hospitalisation, tiredness) during the last 2-weeks during follow-up, and the participants’ results who did not report any health difference over the 2-week interval were used for analysis.

### Data analysis

All analyses described above were performed using IBM Statistical Package for Social Sciences 20, with a level of significance of α = 0.05. The normality of continuous variables was tested using the Kolmogorov–Smirnov test.

Discriminative power was assessed using the independent sample T-test. Internal consistency of the total score and after step wise deletion of each domain was confirmed using Cronbach’s alpha coefficient. Cronbach’s alpha was considered to indicate good reliability at values of 0.7 or more. The correlation among domains and each domain with the total score of the SarQoL®-Kannada questionnaire were assessed using Pearson’s product-moment correlation (r).

Test–retest reliability between the first and the second scores of the SarQoL®-Kannada questionnaire was confirmed using the ICC (two-way mixed, absolute agreement) [18]. ICC was considered to indicate good reliability at values of 0.7 or more [27]. The construct validity with EQ-5D and WHOQOL-BREF was analysed using Spearman’s rank correlation coefficient.

## Results

### SarQoL®-Kannada translation

In phase I (7 days), the translators found six words challenging to translate into Kannada (Supplementary material 6), and the translation of these words was discussed in phase II. Both translators found similar challenges while translating the ambiguous six words into Kannada. For three words to resolve, the Hindi version of the questionnaire was looked into to get the suitable word for the Kannada language (Supplementary material 7).

When the back translated version (phase III, 45 days) was compared with the original English version in phase IV conducted on MS teams online (2 h 30 min), thirteen major and nine minor discrepancies were noted, and those were resolved in phase IV. Word changes were done for eight words, and sentence modifications were done for sixteen sentences, respectively (Supplementary materials 8 and 9).

The pre-final version was tested on nineteen (n = 19) participants. Ten participants (n = 10) were sarcopenic (Mean age 71.8 ± 8.48), and nine (n = 9) were non-sarcopenic (Mean age 68.22 ± 6.14) as per the SARC-F score (sarcopenic: 5.3 ± 1.61 and non-sarcopenic: 1.77 ± 1.22).

After the completion of phase V (40 days), the participants’ suggestions and/or feedback were noted (Supplementary material 10) and discussed with the experts. Four major changes were incorporated in the form of change sentences, while four minor changes were incorporated in the form of changes in words (Supplementary material 11). The summary of modifications done in the questionnaire is summarised in (supplementary material 12).

## Validation results of the SarQoL®-Kannada questionnaire

### Demographic characteristics (n = 114)

A total of one hundred thirty-four (n = 134) participants were screened (Fig. 1). Based on the selection criteria, sarcopenia assessment was conducted for n = 114 participants (sarcopenic participants n = 45, and n = 69 non sarcopenic participants). The mean age of sarcopenic participants (males n = 25; 55.6 % and females n = 20; 44.4 %) was more than the non-sarcopenic participants (males n = 43; 62.3% and females n = 26; 37.7%) (72.22 ± 6.70 vs. 67.88 ± 5.77). There was a significant difference in the SMI, grip strength, and 5-STS among the sarcopenic and non-sarcopenic groups (p < 0.001). The BMI of the sarcopenia group is lower than that of the non-sarcopenia group (23.46 ± 4.25 kg/m^2^ vs. 25.57 ± 3.65 kg/m^2^, p = 0.006) (Table 1).

**Table 1:** Demographic characteristics of the participants

Domain	Sarcopenic (n = 45)	Non-sarcopenic (n = 69)	p value
Age (Years)	72.22 ± 6.70	67.88 ± 5.77	< 0.001
Gender			0.479
Male n (%)	25 (55.6)	43 (62.3)	
Female n (%)	20 (44.4)	26 (37.7)	
Weight (Kg)	59.90 ± 10.44	65.39 ± 10.26	0.007
Height (cm)	160.02 ± 9.02	160.04 ± 8.18	0.989
Body mass index (Kg/m^2^)	23.46 ± 4.25	25.57 ± 3.65	0.006
Education level			0.921
Primary level n (%)	19 (42.3)	30 (43.5)	
Secondary level n (%)	20 (44.4)	28 (40.6)	
Higher level n (%)	6 (13.3)	11 (15.9)	
Skeletal Muscle Index (Kg/m^2^)	5.59 ± 0.86	6.53 ± 0.98	< 0.001
Hand grip strength (Kg)	20.93 ± 7.00	26.87 ± 7.18	< 0.001
5-Sit to Stand (sec)	15.06 ± 3.67	11.35 ± 2.03	< 0.001

### Discriminative power

Sarcopenic participants reported reduced quality of life compared to non-sarcopenic participants (56.43 ± 11.32 vs. 79.38 ± 8.16, p < 0.001), which shows a good discriminative power of the SarQoL®-Kannada questionnaire. Moreover, sarcopenic individuals had significantly lower scores in all domains (Table 2).

**Table 2:** Discriminative power of the SarQoL®-Kannada questionnaire

	Sarcopenic (n = 45) Mean ± SD	Non-sarcopenic (n = 69) Mean ± SD	p value
Total Score	56.43 ± 11.32	79.38 ± 8.16	< 0.001
Domain 1: Physical and Mental Health	52.47 ± 10.85	71.84 ± 10.69	< 0.001
Domain 2: Locomotion	56.42 ± 15.98	79.71 ± 10.40	< 0.001
Domain 3: Body composition	63.60 ± 13.74	78.25 ± 11.45	< 0.001
Domain 4: Functionality	60.10 ± 12.90	83.59 ± 8.46	< 0.001
Domain 5: Activities of Daily Living (ADL)	54.66 ± 15.60	83.46 ± 12.19	< 0.001
Domain 6: Leisure activities	30.69 ± 12.27	38.34 ± 13.45	0.003
Domain 7: Fears	75.00 ± 16.42	89.67 ± 9.81	< 0.001

### Internal consistency

The complete questionnaire showed an alpha of 0.904; the value above 0.70 is indicated as adequate internal consistency with a low risk of redundancy in the questionnaire. Deletions of single domains showed Cronbach’s alpha values ranging from 0.885 to 0.922 (Table 3). Furthermore, the correlations between each domain and the total score of the SarQoL®-Kannada questionnaire were also assessed using Pearson’s coefficients. All domains showed a strong significant positive correlation with the overall score of the SarQoL®-Kannada, except for domain 6 (Table 4).

**Table 3:** Internal consistency reliability of SarQoL®-Kannada questionnaire domains

	Cronbach’s alpha if domain deleted (n = 45)	Overall Cronbach’s alpha
**Total Score**		0.904
Domain 1: Physical and Mental Health	0.889	
Domain 2: Locomotion	0.889	
Domain 3: Body composition	0.885	
Domain 4: Functionality	0.885	
Domain 5: Activities of Daily Living (ADL)	0.888	
Domain 6: Leisure activities	0.922	
Domain 7: Fears	0.894	

**Table 4:** Correlation between individual domain scores of SarQoL®-Kannada questionnaire in sarcopenic participants (n = 45)

	Total score	Domain1	Domain 2	Domain 3	Domain 4	Domain 5	Domain 6	Domain 7
Total score	1							
Domain 1	0.802^**^	1						
Domain 2	0.812^**^	0.640^**^	1					
Domain 3	0.579^**^	0.660^**^	0.386^**^	1				
Domain 4	0.871^**^	0.624^**^	0.668^**^	0.400^**^	1			
Domain 5	0.881^**^	0.592^**^	0.577^**^	0.430^**^	0.670^**^	1		
Domain 6	0.261	0.286	0.054	0.232	0.100	0.278	1	
Domain 7	0.546^**^	0.473^**^	0.357^*^	0.446^**^	0.433^**^	0.438^**^	0.164	1

### Floor and ceiling effect

There was no floor-or ceiling-effect observed, as there was no participant (n = 45) presented with the lowest score on the questionnaire (0 points) or the maximal score (100 points). Also, not more than 15% of the participants had lower or higher scores.

### Construct validity

The results of the construct validity analyses are all presented in Table 5. In general, good correlations were found across the SarQoL®-Kannada with both the EQ-5D-3 L and WHOQOL-BREF questionnaire. When comparing a domain similar to the SarQoL®-Kannada (convergent validity) using the EQ-5D-3 L and WHOQOL-BREF questionnaire, the Spearman’s rho correlations were − 0.51 (p = 0.009) and − 0.50 (p = 0.011) for usual activities and mobility domain of EQ-5D-3 L, while the Pearson’s correlation was 0.50 (p = 0.011) for utility score of EQ-5D-3 L and 0.80 (p < 0.001) for physical health domain of WHOQOL-BREF. When comparing the different domains (divergent validity), a weak correlation was found for the pain/ discomfort (-0.26) and anxiety/depression (-0.18) domain of the EQ-5D-3 L questionnaire. While the moderate strong correlation has been found with three of the domains of the WHOQOL-BREF questionnaire ranging from 0.65 to 0.72 (Table 5).

**Table 5:** Correlation between Total SarQoL®-Kannada questionnaire scores and the EQ-5D-3 L and the WHOQOL-BREF questionnaire

	Total SarQoL scores, r	p value
***Convergent validity***		
**EQ-5D-3 L**		
Utility score	0.50^a^	0.011
Usual activities	-0.51^b^	0.009
Mobility	-0.50^b^	0.011
**WHOQOL-BREF**		
Physical Health	0.80^a^	< 0.001
***Divergent validity***		
**EQ-5D-3 L**		
Self-care	-0.58^b^	0.002
Pain/Discomfort	-0.26^b^	0.210
Anxiety/Depression	-0.18^b^	0.381
**WHOQOL-BREF**		
Psychological	0.65^a^	< 0.001
Social relationship	0.62^a^	0.001
Environment	0.72^b^	< 0.001

^a^Pearson’s product moment correlation (data normally distributed)

^b^Spearman’s rho (data not normally distributed)

### Test-retest reliability

Test-retest reliability was assessed of twenty-five (n = 25) sarcopenic participants. The agreement between the test and retest of the SarQoL®-Kannada overall score was excellent (ICC = 0.97, CI 0.92–0.98). For the individual domains, ICCs ranged from 0.75 to 0.95, with the lowest ICC found for domain 7: fear (ICC = 0.75, CI 0.44–0.89) (Table 6).

**Table 6:** Test retest reliability of the SarQoL®-Kannada questionnaire

	ICC	95% CI
Total Score	0.97	0.92–0.98
Domain 1: Physical and Mental Health	0.88	0.74–0.95
Domain 2: Locomotion	0.90	0.77–0.95
Domain 3: Body composition	0.85	0.68–0.93
Domain 4: Functionality	0.95	0.89–0.97
Domain 5: Activities of Daily Living (ADL)	0.92	0.65–0.97
Domain 6: Leisure activities	0.93	0.85–0.97
Domain 7: Fears	0.75	0.44–0.89

*ICC* intra class correlation coefficient, *CI* confidence interval

## Discussion

This study was conducted with the objective to translate and cross-culturally adapt the SarQoL® questionnaire into Kannada, which is comparable with the original instrument in terms of content and accuracy, and to determine its psychometric properties. The principal finding of this study was that the newly translated SarQoL®-Kannada questionnaire demonstrated itself to be a valid and reliable instrument for measuring the QoL in older people diagnosed with the AWGS 2019 algorithm for sarcopenia. To date, the questionnaire is available online (https://sarqol.org/sites/sarqol/files/SarQoL%20Kannada.pdf).

The results of our study showed that the Kannada version of the original SarQoL® is a valid and discriminant questionnaire that is useful for determining the QoL of patients with sarcopenia. The SarQoL® is the first QoL questionnaire specific to sarcopenia available in the Kannada language. In the 2011 census, people aged 60 and above accounted for 8.6% of the total Indian population and 9.3% only in the state of Karnataka [28]; thus, the SarQoL®-Kannada questionnaire can be a reliable and cost-effective tool for assessing QoL among older patients of Karnataka possibly affected by sarcopenia.

The important thing in old age is not about the length of the remaining life but about the QoL. The QoL of older adults is the ability to achieve a meaningful and satisfying life [29]. QoL assessments via questionnaires are obviously important and necessary for healthcare staff to understand the needs of older people and people with sarcopenia. QoL measures prioritise problems, facilitate communication and monitor changes or responses to treatment. Using the appropriate QoL measure in clinical practice ensures that treatment plans and evaluations focus on the patient rather than the disease [30]. Therefore, developing and refining reliable, valid, user-friendly, standardised ability rating scales is of major importance. Most of the QoL measures are developed & researched in the West and English language, which limits their universal acceptance due to different cultures and languages [31]. Therefore, cultural adaptation of QOL instruments using standard procedures is becoming increasingly important in different countries and across different cultures. This is to ensure the optimal transfer of the original message and measure what is intended to be measured [32]. Unfortunately, there is no tool specifically for the Kannada-speaking sarcopenic population in India to measure the QoL. Thus, in the present study, translation and cross-cultural adaptation of the English SarQoL® questionnaire to Kannada and determining its psychometric properties were done.

The translation of a tool is a time-consuming process that requires numerous discussions to obtain consensus. It involves a lot of individual labour and in-depth group discussions to ensure that consensus decisions led to the most relevant terms being used in the translated instrument [33]. The SarQoL® developer’s translation protocol was strictly followed, in line with universally accepted guidelines [21]. This supports the consistency of a strict translation method to ensure vocabulary equivalence, idiomatic equivalence, and grammatical syntactical equivalence.

Semantic equivalence requires each item or statement to retain its meaning as in the original version, and this turned out to be the biggest challenge. The investigators found that a few questions in the original questionnaire were difficult to translate into Kannada owing to a lack of equivalent words in the native tongue to convey the exact meaning. Difficulties were encountered in translating the following expressions: “DIY (in question no. 3)”, “Washing-up (in question no. 3)”, “Vacuum cleaning (in questions no. 4 & 17)”, “Arm rest (in question no. 17)”, “Banister (in question no. 17)”, “Playing bridge (in question no. 22)”. We used words closest in meaning to communicate the idea.

The study of each item to see whether the notion it measures is relevant to the cultural situation in which it is to be used is referred to as content equivalence [34]. The items: “Choose as many answers as you like (in question no. 7)”, “I feel a weakness in the muscles (in question no. 3; item 3)”, “I’ve had to face the death of several people close to me (in question no. 3; item 5)”, “I do not have much energy, I am often tired (in question no. 3; item 6)”, “My eyesight is poor (in question no. 3; item 7)”, “Do you feel physically weak? (question no. 8)”, “Do you feel you are limited in: (question no. 9)”, “The length of your steps (question no. 9; item 5)”, “Choose as many answers as you like (in question no. 14)”, “Loss of height (in question no. 14; item 3)”, “Loss of muscle mass (in question no. 14; item 4)”, “Hair loss (in question no. 14; item 5)”, “Getting white or grey hair (in question no. 14; item 6)”, “Choose as many answers as you like (in question no. 19)”, “I am not sexually active (in question no. 20; item 1)” proved challenging to translate in the Kannada. The panel deliberated whether to translate the question about sex life “question no. 20 Does your muscle weakness limit your sex life” since it may be offensive to ask elderly or bereaved persons in Indian culture. The expert panel also considered question related to recreational activities “question no. 22 How has your participation in leisure activities (going out to eat, gardening, doing DIY, shooting/fishing, senior citizens clubs, playing bridge, going for a walk, etc.) changed?“ as many activities were not suitable for Indian culture, such as attending to a senior citizens club, playing bridge, and shooting.

Indeed, the literature has recorded that maintaining semantic and content equivalence are the most challenging aspects of translating scientific questionnaires [35, 36]. For the research to progress in a multicultural milieu, it is essential to have culturally adapted research tools that satisfy all the equivalence criteria. The QoL scales are used in allied, psychiatric, social and medical research and are, thus, likely to have a wide utility. This effort at the Kannada translation of the SarQoL® (the disease-specific questionnaire to assess the QoL among sarcopenic participants) is an important step in bringing a locally adapted tool for the benefit of researchers as well the population from this part of the world. Even though cross-cultural adaptation is necessary and has been reported in various documents, the steps involved in it with the minute details are not available. Hence this paper would help the researchers interested in the translation and cross-cultural adaption of outcome measures.

In our study, sarcopenic participants were older and had a lower BMI than non-sarcopenic participants. This is consistent with earlier findings indicating that sarcopenia is associated with older age and lower BMI [37]. Sarcopenic participants had a lower overall score than non-sarcopenic participants. The scores for all domains except domain 6 (leisure activities) were significantly lower in sarcopenic participants. Other translation and validation studies have yielded similar results [10, 12, 14]. As a result, the discriminative power of the SarQoL®-Kannada was confirmed. Within domain 6 (leisure activities), there was no difference between the two groups, which could be explained to some extent by cultural background, as older people in India tend to participate in fewer sports and recreational activities [38]. Only 6.3% of older adults in India, according to a study done in low- and middle-income nations, engage in more than 150 min per week of leisure activity [39]. Other SarQoL® validation studies also showed no difference in the score of domain 6 (leisure activities) between sarcopenic and non-sarcopenic [19, 20].

The overall Cronbach’s alpha of SarQoL®-Kannada was 0.904, suggesting a high internal consistency. A value above 0.70 indicates adequate internal consistency with a low questionnaire redundancy risk [26]. Cronbach’s alpha value remained in the acceptable range with the deletion of subsequent domains, indicating that no domain had a disproportionate influence on the homogeneity of the questionnaire. The correlation matrix showed a significant correlation between the total SarQoL® score and all domains except domain 6. Between domain correlation analysis showed significant correlation except for domain 6.

The convergent validity analyses revealed that the SarQoL®-Kannada questionnaire had significantly good correlations with similar domains of EQ-5D-3 L (utility score, usual activities, mobility) and WHOQOL-BREF (physical health) questionnaires. The divergent validity analyses showed good correlations between an overall score of the SarQoL® and the self-care domain of EQ-5D-3 L, while psychological, social relationship and environment domains of WHOQOL-BREF. However, there was a weak correlation for pain/discomfort and anxiety/depression domains of the EQ-5D-3 L questionnaire, as these features are not central ones in sarcopenia. These results are similar to those in validation studies [12, 14, 20]. However, the WHOQOL-BREF questionnaire has been used in place of Short Form-36 (SF-36) as the Kannada version of the same was not available.

Test–retest reliability was found to be excellent for both the total score and the individual domains of the questionnaire, indicating that the tool’s results are highly reproducible. This trend showed similar results in other SarQoL® validity studies [12–14, 18]. These results were comparable with other validation studies for the total score, which had ICC scores ranging between 0.91 (95% CI 0.82–0.95) and 0.97 (95% CI = 0.95–0.99) [10, 12, 14, 16, 18, 40]. The SarQoL®-Kannada seems to be stable across time when no health changes occurred.

### Strengths and limitations

The strengths of this study include: a large sample size of sarcopenic subjects, the largest recruited so far for any of the SarQoL® validation studies. Also, we used the updated AWGS 2019 criteria, and our results are comparable with other validation studies. The study adhered to the methods recommended for translation and validation of the questionnaire and detailed supplementary material add to the strength. Our study had a few limitations as well. First and foremost, sensitivity to change could not be determined because of the cross-sectional study design. Second, because dual X-ray absorptiometry (DXA) could not be used, we measured muscle mass using BIA, which is less reliable than DXA. On the other hand, BIA has been recognised as a viable instrument for estimating SMI since it is portable, widely available, quick, non-invasive, affordable, and user-friendly. BIA was also used to determine muscle mass in the Dutch and Greek versions of the SarQoL®.

### Future recommendation

The longitudinal and structural validity of the SarQoL®-Kannada is unknown and will need to be studied in future studies. Also, randomized clinical trials can be conducted to evaluate the effect of various programs like multimodal exercise program, reablement program, and nutrition supplementation on the change in quality of life among sarcopenic participants using SarQoL®-Kannada questionnaire.

### Clinical significance

The availability of the validated SarQoL®-Kannada questionnaire gives physicians and researchers speaking this language the chance to better follow and monitor the QoL of sarcopenic patients in Karnataka. Thus, the Kannada version of SarQoL® may be potentially incorporated into the routine geriatric assessment of sarcopenic Kannada-speaking people.

## Conclusion

The SarQoL®-Kannada questionnaire is valid, consistent and reliable for the measurement of quality of life of sarcopenic participants. SarQoL®-Kannada questionnaire is now available to be used in clinical practice and as a treatment outcome indicator in research.

## Electronic supplementary material

Below is the link to the electronic supplementary material.

Supplementary Material 1

Supplementary Material 2

Supplementary Material 3

Supplementary Material 4

Supplementary Material 5

Supplementary Material 6

Supplementary Material 7

Supplementary Material 8

Supplementary Material 9

Supplementary Material 10

Supplementary Material 11

Supplementary Material 12

## Data Availability

The datasets used and/or analysed during the current study are available from the corresponding author on reasonable request.
