# Supplementary material for: Psychometric performance of the Kannada version of sarcopenia quality of life questionnaire (SarQoL®)
Source: BMC Musculoskelet Disord. 2023 Jun 2;24:445. doi: 10.1186/s12891-023-06559-8 (PMC10236591; doi:10.1186/s12891-023-06559-8)
Supplement: Supplementary file 1 — Supplementary Material 1 [file 12891_2023_6559_MOESM1_ESM.pdf]

Phase 1: Initial translations (English to Kannada)

In that SarQoL questionnaire it has been found that some phrases/words which couldn't be translated easily in kannada like DIY (in question. no 3) which has no exact word for the same. So, it was written as the phrase “ನಿಮಗೆ ಇಷ್ಟವಾದ ಕೆಲಸವನ್ನು ಮಾಡುವುದು (Nimage iṣṭavāda kelasavannu māḍuvudu”. For vacuum cleaning (in question no. 4 & 17) there is phrase like “nirvata suchigolivike (ನಿರ್ವಾತ ಶುಚಿಗೊಳಿವಿಕೆ)” but generally people do not use the word in day-to-day life so same phrase as “ವ್ಯಾಕ್ಯುಮ್ ಕ್ಲೀನಿಂಗ್ (Vyākyum klīniṅg)” in Kannada script have been used. Also, couldn’t find Kannada word for arm rest (in question no. 17), Banister (in question no. 17) and playing bridge (in question no. 22) hence they were written as “ಆರ್ಮ್ ರೆಸ್ಟ್ (Ārm resṭ)”, “ಬ್ಯಾನಿಸ್ಟರ್ (Byānisṭar)” and “ಕಾರ್ಡ್ (ಇಸ್ಪೀಟ್) ಆಡುವುದು (Kārḍ (ispīṭ) āḍuvudu)”. In question no. 3 for the word washing-up there was a confusion so the Hindi SarQol questionnaire were referred where it has been written as “स्नान आदि (snaan aadi)” accordingly in kannada “ಸ್ನಾನ ಮಾಡುವುದು (Snāna māḍuvudu)”.
