# Supplementary material for: Psychometric performance of the Kannada version of sarcopenia quality of life questionnaire (SarQoL®)
Source: BMC Musculoskelet Disord. 2023 Jun 2;24:445. doi: 10.1186/s12891-023-06559-8 (PMC10236591; doi:10.1186/s12891-023-06559-8)
Supplement: Supplementary file 2 — Supplementary Material 2 [file 12891_2023_6559_MOESM2_ESM.pdf]

Phase 2: Synthesis

After discussing with the second translator, we prepared the translated Version 1 of the questionnaire. We both found similar difficulties while translating the questionnaire. They are as follows: In question number 3 and 22, for the word “DIY” there is no particular kannada word. So, we have written “ನಿಮಗೆ ಇಷ್ಟವಾದ ಕೆಲಸವನ್ನು ಮಾಡುವುದು” (Nimage iṣṭavāda kelasavannu māḍuvudu) which gives similar meaning. In question number 4 and 17 for the word “hoovering” we referred to Hindi questionnaire to understand the meaning. There they have written “वैक्यूम क्लीनिंग”. In kannada there is phrase like “ನಿರ್ವಾತ ಶುಚಿಗೊಳಿಸುವಿಕೆ “. Generally, people cannot understand that much kannada, so we used same phrase as “ವ್ಯಾಕ್ಯೂಮ್ ಕ್ಲೀನಿಂಗ್” in kannada script. In question number 17, for the word “Banister” we used Hindi questionnaire to find the meaning. There they have written “रेलिंग”. In kannada there is no particular word for Banister/ Handrail, so we used same word “ಬ್ಯಾನಿಸ್ಟರ್ (ಹ್ಯಾಂಡ್ ರೇಲ್) in kannada script. In question number 22 for the word “playing bridge” even in Hindi questionnaire they translated same word to Hindi script as “ब्रिज खेलना” so we searched on internet and found the meaning as “playing cards” so we have written it as “ಕಾರ್ಡ್ಸ್ (ಇಸ್ಪೀಟ್) ಆಡುವುದು “.
