# Supplementary material for: Psychometric performance of the Kannada version of sarcopenia quality of life questionnaire (SarQoL®)
Source: BMC Musculoskelet Disord. 2023 Jun 2;24:445. doi: 10.1186/s12891-023-06559-8 (PMC10236591; doi:10.1186/s12891-023-06559-8)
Supplement: Supplementary file 3 — Supplementary Material 3 [file 12891_2023_6559_MOESM3_ESM.pdf]

Expert Committee Meeting Report

The expert committee meeting was organised on 3rd of August 2022.

Under the guidance of expert committee members, we prepared the final questionnaire by making the following changes:

In question no. 1, for the option “ಕೆಲವು” we changed the word as “ಸ್ವಲ್ಪ ಮಟ್ಟಿಗೆ ". For question no. 3 instead of “ಲಘುವಾದ” we changed the word as “ಹಗುರವಾದ” and we removed the word “ಇವುಗಳಲ್ಲಿ”. In question no. 4 we removed the word “ಕೀಳುವುದು” which was written in brackets and the word “ಇವುಗಳಲ್ಲಿ” from question no. 4 and 5. Instead of “ಮಧ್ಯಮ” we changed the word as “ಕಷ್ಟಕರವಲ್ಲದ”. In question no. 7, We changed the sentence which was written in brackets “ಈ ಕೆಳಗಿನ ಆಯ್ಕೆಗಳಲ್ಲಿ ನಿಮಗೆ ಇಷ್ಟವಾದ ಒಂದಕ್ಕಿಂತ ಹೆಚ್ಚಿನ ಆಯ್ಕೆಗಳನ್ನು ಆಯ್ದುಕೊಳ್ಳಬಹುದು” as “ನೀವು ಇಷ್ಟಪಡುವಷ್ಟು ಉತ್ತರಗಳನ್ನು ಆಯ್ದುಕೊಳ್ಳಬಹುದು”. And for the option 3, for the sentence “ನಾನು ಸ್ನಾಯುಗಳಲ್ಲಿ ದೌರ್ಬಲ್ಯವನ್ನು ಹೊಂದಿದ್ದೇನೆ” changed as “ನನ್ನ ಸ್ನಾಯುಗಳು ದುರ್ಬಲವಾಗಿವೆ ಎಂದು ಭಾವಿಸುತ್ತೇನೆ”. In the option 5, instead of writing “ನನ್ನ ಹತ್ತಿರದವರ ಹಲವಾರು ಜನರ ಸಾವನ್ನು ನಾನು ಎದುರಿಸಿರುತ್ತೇನೆ” changed as “ನನ್ನ ಹತ್ತಿರದವರ ಹಲವಾರು ಜನರ ಸಾವನ್ನು ನೋಡಿದ್ದೇನೆ”. In the option 6, we changed the sentence “ನಾನು ಶಕ್ತಿಹೀನನಾಗಿರುತ್ತೇನೆ” as “ನನಗೆ ಶಕ್ತಿಯಿಲ್ಲ”. In the option 7, “ನಾನು ಕಳಪೆ ದೃಷ್ಟಿಯನು ಹೊಂದಿದ್ದೇನೆ” as “ನನ್ನ ಕಣ್ಣಿನ ದೃಷ್ಟಿ ಮಂದವಾಗಿದೆ”. For question no.8 we changed the question “ನೀವು ದೈಹಿಕವಾಗಿ ದೌರ್ಬಲ್ಯವನ್ನು ಅನುಭವಿಸುತ್ತಿದ್ದೀರಾ?” as “ನೀವು ದೈಹಿಕವಾಗಿ ದುರ್ಬಲರಾಗಿದ್ದೀರಾ?”. We changed the question no. 9 “ಇವುಗಳಲ್ಲಿ ನೀವು ಸೀಮಿತರೆಂದು ಭಾವಿಸುತ್ತೀರಾ?” as “ಈ ಕೆಳಗಿನ ಚಟುವಟಿಕೆಗಳಲ್ಲಿ”. And for the option 5 after the word “ಹೆಜ್ಜೆ ಇಟ್ಟು” we have added “ಹೆಜ್ಜೆಗಳ ನಡುವಿನ ಅಂತರ” within brackets. For question

no. 13 instead of word “ದೈಹಿಕ ರೂಪದಲ್ಲಿ” we changed the word as “ದೇಹದ ಆಕಾರದಲ್ಲಿ”. We changed the question no. 14 which was written in brackets “ಈ ಕೆಳಗಿನವುಗಳಲ್ಲಿ ಒಂದಕ್ಕಿಂತ ಹೆಚ್ಚಿನ ಆಯ್ಕೆಗಳನ್ನು ಆಯ್ದುಕೊಳ್ಳಬಹುದು” as “ಈ ಕೆಳಗಿನವುಗಳಲ್ಲಿ ನಿಮ್ಮ ಆಯ್ಕೆಗಳನ್ನು ಆಯ್ದುಕೊಳ್ಳಬಹುದು”. We changed the option 3 “ಎತ್ತರದ ನಷ್ಟವಾಗಿದೆ” as “ಎತ್ತರ ಕಡಿಮೆಯಾಗಿದೆ”. In the option 4, “ಸ್ನಾಯು (ಮಾಂಸ ಖಂಡ) ವಿನ ಬಲವನ್ನು ಕಳೆದುಕೊಂಡಿದ್ದೇನೆ” as “ಮಾಂಸಖಂಡಗಳು (ಸ್ನಾಯುಗಳು) ಕ್ಷೀಣಿಸುತ್ತಿವೆ”. We changed the option 5, “ಕೂದಲು ಉದುರುವಿಕೆಯನ್ನು ಹೊಂದಿದ್ದೇನೆ” as “ಕೂದಲು ಉದುರುತ್ತಿದೆ”. In the option 6, “ ಬಿಳಿ ಅಥವಾ ಬೂದು ಬಣ್ಣದ ಕೂದಲನ್ನು ಹೊಂದಿದ್ದೇನೆ” as “ ಕೂದಲು ಬಿಳಿಯಾಗುತ್ತಿದೆ”. For question no.15, We have added one word “ನೀವು” in between the sentence. In question no. 17, for option 4, the word “squating “is written as “ಕೆಳಗೆ ಕುಳಿತುಕೊಳ್ಳುವುದು”. In option 5 “ಬಗ್ಗಿ ತೆಗೆಯುವುದು”is changed to “ಎತ್ತಿಕೊಳ್ಳುವುದು”. In option 6 we have added “ಕೈಗಳಿಲ್ಲದ (ಆರ್ಮ್ ರೆಸ್ಟ್). We removed the word “ವಸ್ತುಗಳಿಂದ ತುಂಬಿದ” and “ಅನ್ನು” from option 9 and 14 respectively. We changed the word “ಮಿತಗೊಳಿಸುತ್ತಿದೆಯೇ? as “ ಅಡ್ಡಿ ಗೊಳಿಸುತ್ತಿದ್ದೆಯೇ?” in question no. 18. For question no 19 the sentence which was written in brackets “ನಿಮಗೆ ಇಷ್ಟವಾದಂತಹ ಒಂದಕ್ಕಿಂತ ಹೆಚ್ಚಿನ ಆಯ್ಕೆಯನ್ನು ಆಯ್ದುಕೊಳ್ಳಬಹುದು” as “ನಿಮ್ಮ ಆಯ್ಕೆಗಳನ್ನು ಆಯ್ದುಕೊಳ್ಳಬಹುದು”. In question no. 19, for option 1 we have added “ಲೈಂಗಿಕವಾಗಿ ಸಕ್ರಿಯವಾಗಿಲ್ಲ” in brackets. We changed the word “ಬದಲಾವಣೆಯಾಗಿದೆ?” as " ಬದಲಾಗಿದೆ” in question no. 21. And for last question (question no.22) we changed the question as “ ನಿಮ್ಮ ಬಿಡುವಿನ (ವಿರಾಮದ) ಸಮಯದ ಚಟುವಟಿಕೆಗಳಲ್ಲಿ (------) ನಿಮ್ಮ ಭಾಗವಹಿಸುವಿಕೆ ಹೇಗೆ ಬದಲಾಗಿದೆ ?
