# Supplementary material for: Psychometric performance of the Kannada version of sarcopenia quality of life questionnaire (SarQoL®)
Source: BMC Musculoskelet Disord. 2023 Jun 2;24:445. doi: 10.1186/s12891-023-06559-8 (PMC10236591; doi:10.1186/s12891-023-06559-8)
Supplement: Supplementary file 4 — Supplementary Material 4 [file 12891_2023_6559_MOESM4_ESM.pdf]

Phase 5: Test of pre-final version

After giving this questionnaire to some older adults they have suggested some changes in the questionnaire so we have made those changes after discussing with expert committee members those are as follows:

Wherever the word ಸ್ನಾಯು (Muscle) is there we added one more alternative word as ಮಾಂಸಖಂಡ in brackets. And we removed the word ವ್ಯಾಕ್ಯೂಮ್ ಕ್ಲೀನಿಂಗ್ and added "ಗುಡಿಸುವುದು"," ಒರೆಸುವುದು". In question no. 1, instead of "ತೋಳುಗಳಲ್ಲಿನ" we changed the word as "ಕೈಗಳಲ್ಲಿನ". We explained the meaning of word "ನಮ್ಯತೆ" (flexibility) in bracket. For question no. 4 instead of "ಕಷ್ಟಕರವಲ್ಲದ" we changed the word as ತುಂಬಾ ಕಷ್ಟವಲ್ಲದ and we changed the sentence "ತೋಟದಲ್ಲಿನ ಕಳೆಯನ್ನು ತೆಗೆಯುವುದು" as "ತೋಟದಲ್ಲಿನ ಹುಲ್ಲು ಅಥವಾ ಬೇಡವಾದ ಗಿಡಗಳನ್ನು ತೆಗೆಯುವುದು" In question no. 6 We changed the word "ಪ್ರಸ್ತುತ" as "ಈಗ". For question no.9 we changed the order of option from "ತುಂಬಾ, ಸ್ವಲ್ಪ, ಕಡಿಮೆ, ಇಲ್ಲ " to "ಇಲ್ಲ, ಕಡಿಮೆ, ಸ್ವಲ್ಪ, ತುಂಬಾ". In question no.11 for the word "ಸಮತೋಲನ " (Balance) we added explanation for the same in brackets. In question no. 17, instead of "ಬ್ಯಾನಿಸ್ಟರ್ (ಹ್ಯಾಂಡ್ ರೇಲ್) ಅನ್ನು ಹಿಡಿದುಕೊಳ್ಳದೆ ಒಂದು ಅಥವಾ ಹಲವಾರು ಮಹಡಿಯ ಮೆಟ್ಟಿಲನ್ನು ಹತ್ತುವುದು we changed the sentence as "ಮೆಟ್ಟಿಲು ಹತ್ತುವಾಗ ಗೋಡೆ ಅಥವಾ ಬೇರೆ ಯಾವುದರ ಸಹಾಯವಿಲ್ಲದೆ ಒಂದು ಅಥವಾ ಹಲವಾರು ಮೆಟ್ಟಿಲುಗಳನ್ನು ಹತ್ತುವುದು" and instead of "ಕಡಿಮೆ ಎತ್ತರವಿರುವ ಕೈಗಳಿಲ್ಲದೆ (ಆರ್ಮ್ ರೆಸ್ಟ್) ಇರುವ ಕುರ್ಚಿಯಿಂದ ಮೇಲೇಳುವುದು" changed the sentence as " ಕಡಿಮೆ ಎತ್ತರ ಇರುವ ಕೈಗಳಿಲ್ಲದ ಅಥವಾ ಹಿಡಿಯಲು ಏನೂ ಇಲ್ಲದ ಕುರ್ಚಿಯಿಂದ ಏಳುವುದು" In question no. 22. We removed the examples " ಶೂಟಿಂಗ್/ ಮೀನು ಹಿಡಿಯುವುದು and added " ಸಮಾರಂಭಗಳಲ್ಲಿ ಭಾಗವಹಿಸುವುದು" and "ಮಕ್ಕಳೊಂದಿಗೆ ಆಟ ಆಡುವುದು"
