# Supplementary material for: Psychometric performance of the Kannada version of sarcopenia quality of life questionnaire (SarQoL®)
Source: BMC Musculoskelet Disord. 2023 Jun 2;24:445. doi: 10.1186/s12891-023-06559-8 (PMC10236591; doi:10.1186/s12891-023-06559-8)
Supplement: Supplementary file 5 — Supplementary Material 5 [file 12891_2023_6559_MOESM5_ESM.pdf]

SarQoL
Sarcopenia and Quality of Life

ಸಾರ್ಕೋಪೆನಿಯಾದಲ್ಲಿ ಜೀವನದ ಗುಣಮಟ್ಟ

ಈ ಪ್ರಶ್ನಾವಳಿಯು ಸಾರ್ಕೋಪೆನಿಯಾ ಅಂದರೆ ವಯಸ್ಸಾಗುತ್ತಿದ್ದಂತೆ ಬರುವ ಸ್ನಾಯುವಿನ (ಮಾಂಸ ಖಂಡಗಳ) ದುರ್ಬಲತೆ ಬಗ್ಗೆ ಕೇಳುತ್ತದೆ. ಈ ಸ್ನಾಯುವಿನ (ಮಾಂಸಖಂಡದ) ದುರ್ಬಲತೆಯು ನಿಮ್ಮ ದೈನಂದಿನ ಜೀವನದ ಮೇಲೆ ಪರಿಣಾಮ ಬೀರಬಹುದು. ಈ ಸಮೀಕ್ಷೆಯು ಹೇಗೆ ನಿಮ್ಮ ಸ್ನಾಯುವಿನ (ಮಾಂಸಖಂಡದ) ಸ್ಥಿತಿ ಜೀವನದ ಗುಣಮಟ್ಟದ ಮೇಲೆ ಪರಿಣಾಮ ಬೀರುತ್ತದೆ ಎಂದು ತಿಳಿಯಲು ಅನುವುಮಾಡಿಕೊಡುತ್ತದೆ.

ದಯವಿಟ್ಟು ಪ್ರತಿ ಪ್ರಶ್ನೆಗೆ ಹೆಚ್ಚು ಸೂಕ್ತವಾದ ಉತ್ತರವನ್ನು ಆಯ್ಕೆಮಾಡಿ. ಈ ಪ್ರಶ್ನಾವಳಿಯನ್ನು ಪೂರ್ಣಗೊಳಿಸಲು ನಿಮಗೆ ಅಂದಾಜು ೧೦ ನಿಮಿಷಗಳು ಬೇಕಾಗಬಹುದು.

1. ಪ್ರಸ್ತುತ ನಿಮಗೆ ಈ ಕೆಳಗಿನವುಗಳಲ್ಲಿ ಯಾವುದಾದರೂ ಕಡಿಮೆಯಾಗಿದೆಯೇ ಎಂದು ಅನಿಸುತ್ತಿದೆಯೇ:

	ತುಂಬಾ	ಸ್ವಲ್ಪಮಟ್ಟಿಗೆ	ಸ್ವಲ್ಪ	ಇಲ್ಲ
ನಿಮ್ಮ ಕೈಗಳಲ್ಲಿನ ಶಕ್ತಿ?	☐	☐	☐	☐
ನಿಮ್ಮ ಕಾಲುಗಳ ಶಕ್ತಿ?	☐	☐	☐	☐
ಸ್ನಾಯು (ಮಾಂಸಖಂಡಗಳ) ಶಕ್ತಿ?	☐	☐	☐	☐
ನಿಮ್ಮ ಸಾಮರ್ಥ್ಯ?	☐	☐	☐	☐
ನಿಮ್ಮ ದೈಹಿಕ ಸಾಮರ್ಥ್ಯಗಳು?	☐	☐	☐	☐
ನಿಮ್ಮ ಸ್ನಾಯುವಿನ (ಮಾಂಸಖಂಡದ) ನಮ್ಯತೆ?<br>[ನಿಮ್ಮ ಮಾಂಸ ಖಂಡಗಳು ಸುಲಭವಾಗಿ ದೇಹವನ್ನು ಬಗ್ಗಿಸಲು ಮತ್ತು ಕೈಕಾಲುಗಳನ್ನು ಮಡಚಲು ಮತ್ತು ಚಾಚಲು ಸಹಕರಿಸುತ್ತಿವೆಯೆ?]	☐	☐	☐	☐

2. ನಿಮ್ಮ ಸ್ನಾಯು (ಮಾಂಸಖಂಡ) ಗಳಲ್ಲಿ ನೋವು ಇದೆಯೇ?

- ☐ ಆಗಾಗ
- ☐ ಕೆಲವೊಮ್ಮೆ
- ☐ ವಿರಳವಾಗಿ (ಅಪರೂಪಕ್ಕೆ)
- ☐ ಎಂದಿಗೂ ಇಲ್ಲ

Copyright© - January 2015 - All right Reserved

3. ನೀವು ಹಗುರವಾದ ದೈಹಿಕ ಚಟುವಟಿಕೆಗಳನ್ನು ಮಾಡುವಾಗ (ನಿಧಾನವಾಗಿ ನಡೆಯುವುದು, ಇಸ್ತ್ರಿಯನ್ನು ಮಾಡುವುದು, ಧೂಳನ್ನು ತೆಗೆಯುವುದು, ಸ್ನಾನ ಮಾಡುವುದು, ನಿಮಗೆ ಇಷ್ಟವಾದ ಕೆಲಸವನ್ನು ಮಾಡುವುದು, ಗಿಡಗಳಿಗೆ ನೀರು ಹಾಕುವುದು ಇತ್ಯಾದಿ) ನಿಮಗೆ ಯಾವಾಗ

	ಆಗಾಗ	ಒಮ್ಮೊಮ್ಮೆ	ಅಪರೂಪಕ್ಕೆ	ಎಂದಿಗೂ ಇಲ್ಲ	ಇಂತಹ ಯಾವುದೇ ಚಟುವಟಿಕೆಗಳನ್ನು ನಾನು ಮಾಡುವುದಿಲ್ಲ
ಕಷ್ಟವಾಗುತ್ತದೆ?	☐	☐	☐	☐	☐
ಸುಸ್ತಾಗುತ್ತದೆ?	☐	☐	☐	☐	☐
ನೋವಿನ ಅನುಭವವಾಗುತ್ತದೆ?	☐	☐	☐	☐	☐

4. ನೀವು ತುಂಬಾ ಕಷ್ಟವಲ್ಲದ ಚಟುವಟಿಕೆಗಳನ್ನು ಮಾಡುವಾಗ (ವೇಗವಾಗಿ ನಡೆಯುವುದು, ಕಿಟಿಕಿಗಳನ್ನು ಸ್ವಚ್ಛಗೊಳಿಸುವುದು, ಗುಡಿಸುವುದು, ಒರೆಸುವುದು,ಕಾರನ್ನು ತೊಳೆಯುವುದು, ತೋಟದಲ್ಲಿನ ಹುಲ್ಲು ಅಥವಾ ಬೇಡವಾದ ಗಿಡಗಳನ್ನು ತೆಗೆಯುವುದು ಇತ್ಯಾದಿ) ನಿಮಗೆ ಯಾವಾಗ

	ಆಗಾಗ	ಒಮ್ಮೊಮ್ಮೆ	ಅಪರೂಪಕ್ಕೆ	ಎಂದಿಗೂ ಇಲ್ಲ	ಇಂತಹ ಯಾವುದೇ ಚಟುವಟಿಕೆಗಳನ್ನು ನಾನು ಮಾಡುವುದಿಲ್ಲ
ಕಷ್ಟವಾಗುತ್ತದೆ?	☐	☐	☐	☐	☐
ಸುಸ್ತಾಗುತ್ತದೆ?	☐	☐	☐	☐	☐
ನೋವಿನ ಅನುಭವವಾಗುತ್ತದೆ?	☐	☐	☐	☐	☐

5. ನೀವು ತೀವ್ರವಾದ ದೈಹಿಕ ಚಟುವಟಿಕೆಗಳನ್ನು ಮಾಡುವಾಗ (ಓಡುವುದು, ತುಂಬಾ ದೂರ ನಡೆಯುವುದು, ಭಾರವಾದ ವಸ್ತುಗಳನ್ನು ಎತ್ತುವುದು, ಪೀಠೋಪಕರಣಗಳನ್ನು ಸರಿಸುವುದು, ತೋಟಗಳನ್ನು ಅಗೆಯುವುದು ಇತ್ಯಾದಿ) ನಿಮಗೆ ಯಾವಾಗ

	ಆಗಾಗ	ಒಮ್ಮೊಮ್ಮೆ	ಅಪರೂಪಕ್ಕೆ	ಎಂದಿಗೂ ಇಲ್ಲ	ಇಂತಹ ಯಾವುದೇ ಚಟುವಟಿಕೆಗಳನ್ನು ನಾನು ಮಾಡುವುದಿಲ್ಲ
ಕಷ್ಟವಾಗುತ್ತದೆ?	☐	☐	☐	☐	☐
ಸುಸ್ತಾಗುತ್ತದೆ?	☐	☐	☐	☐	☐
ನೋವಿನ ಅನುಭವವಾಗುತ್ತದೆ?	☐	☐	☐	☐	☐

6. ಈಗ ನಿಮಗೆ ವಯಸ್ಸಾಗುತ್ತಿದೆ ಎಂದು ಭಾವಿಸುವಿರಾ?

- ☐ ಹೌದು, ತುಂಬಾ
- ☐ ಹೌದು, ಸ್ವಲ್ಪಮಟ್ಟಿಗೆ
- ☐ ಹೌದು, ಸ್ವಲ್ಪ
- ☐ ಇಲ್ಲ, ಇಲ್ಲವೇ ಇಲ್ಲ

Copyright© - January 2015 - All right Reserved

7. 6 ನೇ ಪ್ರಶ್ನೆಗೆ ಉತ್ತರ "ಹೌದು" ಎಂದಾದರೆ, ನಿಮಗೆ ಹಾಗೆ ಏಕೆ ಅನಿಸುತ್ತಿದೆ?

(ನೀವು ಇಷ್ಟಪಡುವಷ್ಟು ಉತ್ತರಗಳನ್ನು ಆಯ್ದುಕೊಳ್ಳಬಹುದು)

- ☐ ನಾನು ಸುಲಭವಾಗಿ ಅನಾರೋಗ್ಯಕ್ಕೆ ತುತ್ತಾಗುತ್ತೇನೆ
- ☐ ನಾನು ಅನೇಕ ಔಷಧಗಳನ್ನು ತೆಗೆದುಕೊಳ್ಳುತ್ತೇನೆ
- ☐ ನನ್ನ ಸ್ನಾಯುಗಳು (ಮಾಂಸಖಂಡ) ದುರ್ಬಲವಾಗಿವೆ ಎಂದು ಭಾವಿಸುತ್ತೇನೆ
- ☐ ನಾನು ಮರೆವಿನ ಸಮಸ್ಯೆಯನ್ನು ಹೊಂದಿದ್ದೇನೆ
- ☐ ನನ್ನ ಹತ್ತಿರದವರ ಹಲವಾರು ಜನರ ಸಾವನ್ನು ನೋಡಿದ್ದೇನೆ
- ☐ ನನಗೆ ಹೆಚ್ಚು ಶಕ್ತಿ ಇಲ್ಲ, ನನಗೆ ಆಗಾಗ ಸುಸ್ತಾಗುತ್ತದೆ
- ☐ ನನ್ನ ಕಣ್ಣಿನ ದೃಷ್ಟಿ ಮಂದವಾಗಿದೆ
- ☐ ಇತರೆ:

8. ನೀವು ದೈಹಿಕವಾಗಿ ದುರ್ಬಲರಾಗಿದ್ದೀರಾ?

- ☐ ಹೌದು, ಸಂಪೂರ್ಣವಾಗಿ
- ☐ ಹೌದು, ಸ್ವಲ್ಪಮಟ್ಟಿಗೆ
- ☐ ಹೌದು, ಸ್ವಲ್ಪ
- ☐ ಇಲ್ಲ, ಇಲ್ಲವೇ ಇಲ್ಲ

9. ಈ ಕೆಳಗಿನ ಯಾವ ಚಟುವಟಿಕೆ ಕಡಿಮೆಯಾಗಿದೆ (ಸೀಮಿತ) ಎಂದು ಭಾವಿಸುತ್ತೀರಿ:

	ತುಂಬಾ	ಕೆಲವು	ಸ್ವಲ್ಪ	ಇಲ್ಲ
ನೀವು ಎಷ್ಟು ಸಮಯ ನಡೆಯಬಲ್ಲಿರಿ?	☐	☐	☐	☐
ನೀವು ಎಷ್ಟು ಬಾರಿ ನಡೆದುಕೊಂಡು ಹೋಗಬಲ್ಲಿರಿ?	☐	☐	☐	☐
ನೀವು ಎಷ್ಟು ದೂರ ನಡೆಯಬಲ್ಲಿರಿ?	☐	☐	☐	☐
ನೀವು ಎಷ್ಟು ವೇಗವಾಗಿ ನಡೆಯಬಲ್ಲಿರಿ?	☐	☐	☐	☐
ನೀವು ಎಷ್ಟು ದೂರ ಹೆಜ್ಜೆ ಇಟ್ಟು (ಹೆಜ್ಜೆಗಳ ನಡುವಿನ ಅಂತರ) ನಡೆಯಬಲ್ಲಿರಿ?	☐	☐	☐	☐

10. ನೀವು ನಡೆಯುವಾಗ (ವಾಕಿಂಗ್ ಮಾಡುವಾಗ):

	ಆಗಾಗ	ಒಮ್ಮೊಮ್ಮೆ	ಅಪರೂಪಕ್ಕೆ	ಎಂದಿಗೂ ಇಲ್ಲ	ನನ್ನಿಂದ ನಡೆಯಲು ಸಾಧ್ಯವಿಲ್ಲ
ನಿಮಗೆ ತುಂಬಾ ಆಯಾಸದ ಅನುಭವವಾಗುತ್ತದೆಯೇ?	☐	☐	☐	☐	☐
ಚೇತರಿಸಿಕೊಳ್ಳಲು ನೀವು ಆಗಾಗ ಕುಳಿತುಕೊಳ್ಳಬೇಕಾಗುತ್ತದೆಯೇ?	☐	☐	☐	☐	☐
ನೀವು ರಸ್ತೆಗಳನ್ನು ವೇಗವಾಗಿ ದಾಟಲು ಕಷ್ಟಪಡುತ್ತೀರಾ?	☐	☐	☐	☐	☐
ನೀವು ಸಮತಟ್ಟಲ್ಲದ ಜಾಗದಲ್ಲಿ ನಡೆಯಲು ತೊಂದರೆಯನ್ನು ಹೊಂದಿರುವಿರಾ?	☐	☐	☐	☐	☐

Copyright© - January 2015 - All right Reserved

11. ನಿಮ್ಮ ಸಮತೋಲನದಲ್ಲಿ (ಬ್ಯಾಲೆನ್ಸ್ – ನೇರವಾಗಿ ನಿಲ್ಲುವಾಗ ಅಥವಾ ನಡೆಯುವಾಗ ಒಮ್ಮೆಗೆ ಬೀಳುವಂತೆ ಆಗುತ್ತದೆಯೇ?) ಸಮಸ್ಯೆಯನ್ನು ಹೊಂದಿರುವಿರಾ?

- ☐ ಆಗಾಗ
- ☐ ಒಮ್ಮೊಮ್ಮೆ
- ☐ ಅಪರೂಪಕ್ಕೆ
- ☐ ಎಂದಿಗೂ ಇಲ್ಲ

12. ನೀವು ಎಷ್ಟು ಬಾರಿ ಬೀಳುತ್ತೀರಿ?

- ☐ ಬಹಳಷ್ಟು ಸಲ
- ☐ ಒಮ್ಮೊಮ್ಮೆ
- ☐ ಅಪರೂಪಕ್ಕೆ
- ☐ ಎಂದಿಗೂ ಇಲ್ಲ

13. ನೀವು ನಿಮ್ಮ ದೇಹದ ಆಕಾರದಲ್ಲಿ ಬದಲಾವಣೆಯಾಗಿದೆ ಎಂದು ಭಾವಿಸುತ್ತೀರಾ?

- ☐ ಹೌದು, ತುಂಬಾ
- ☐ ಹೌದು, ಸ್ವಲ್ಪಮಟ್ಟಿಗೆ
- ☐ ಹೌದು, ಸ್ವಲ್ಪ
- ☐ ಇಲ್ಲ, ಇಲ್ಲವೇ ಇಲ್ಲ

14. 13 ನೇ ಪ್ರಶ್ನೆಗೆ ಉತ್ತರ "ಹೌದು" ಎಂದಾದರೆ, ಅದು ಯಾವ ರೀತಿಯಲ್ಲಿ?
(ನೀವು ಇಷ್ಟಪಡುವಷ್ಟು ಉತ್ತರಗಳನ್ನು ಆಯ್ದುಕೊಳ್ಳಬಹುದು)

- ☐ ನಿಮ್ಮ ತೂಕದಲ್ಲಿನ ಬದಲಾವಣೆ (ನೀವು ತೂಕವನ್ನು ಹೆಚ್ಚಿಸಿದ್ದೀರಿ/ ನೀವು ತೂಕವನ್ನು ಕಳೆದುಕೊಂಡಿದ್ದೀರಿ)
- ☐ ನಿಮ್ಮ ಚರ್ಮದ ಸುಕ್ಕುಗಟ್ಟುವಿಕೆ
- ☐ ಎತ್ತರ ಕಡಿಮೆಯಾಗಿದೆ
- ☐ ಸ್ನಾಯುಗಳು (ಮಾಂಸಖಂಡಗಳು) ಕ್ಷೀಣಿಸುತ್ತಿವೆ
- ☐ ಕೂದಲು ಉದುರುತ್ತಿದೆ
- ☐ ಕೂದಲು ಬಿಳಿಯಾಗುತ್ತಿದೆ
- ☐ ಇತರೆ:

15. 13 ನೇ ಪ್ರಶ್ನೆಗೆ ಉತ್ತರ "ಹೌದು" ಎಂದಾದರೆ, ನೀವು ಈ ಬದಲಾವಣೆಯಿಂದ ಚಿಂತಿತರಾಗಿದ್ದೀರಾ?

- ☐ ಹೌದು, ತುಂಬಾ
- ☐ ಹೌದು, ಸ್ವಲ್ಪಮಟ್ಟಿಗೆ
- ☐ ಹೌದು, ಸ್ವಲ್ಪ
- ☐ ಇಲ್ಲ, ಇಲ್ಲವೇ ಇಲ್ಲ

Copyright© - January 2015 - All right Reserved

16. ನೀವು ದುರ್ಬಲರಾಗಿದ್ದೀರಿ ಎಂದು ಭಾವಿಸುತ್ತೀರಾ?

- ☐ ಖಂಡಿತವಾಗಿ ಹೌದು
- ☐ ಸ್ವಲ್ಪ
- ☐ ಇಲ್ಲವೇ ಇಲ್ಲ

17. ನಿಮಗೆ ಈಗ ಈ ಕೆಳಗಿನ ಯಾವ ದೈನಂದಿನ ಚಟುವಟಿಕೆಗಳನ್ನು ಮಾಡಲು ಕಷ್ಟ ಅನಿಸುತ್ತದೆ?

	ಹಾಗೆ ಮಾಡಲು ಸಾಧ್ಯವಿಲ್ಲ	ತುಂಬಾ ತೊಂದರೆ	ಸ್ವಲ್ಪ ತೊಂದರೆ	ತೊಂದರೆ ಇಲ್ಲ	ಅನ್ವಯಿಸುವುದಿಲ್ಲ
ಒಂದು ಮಹಡಿಯ ಮೆಟ್ಟಿಲನ್ನು ಹತ್ತುವುದು	☐	☐	☐	☐	☐
ಒಂದಕ್ಕಿಂತ ಹೆಚ್ಚು ಮಹಡಿಯ ಮೆಟ್ಟಿಲನ್ನು ಹತ್ತುವುದು	☐	☐	☐	☐	☐
ಮೆಟ್ಟಿಲು ಹತ್ತುವಾಗ ಗೋಡೆ ಅಥವಾ ಬೇರೆ ಯಾವುದರ ಸಹಾಯವಿಲ್ಲದೆ ಒಂದು ಅಥವಾ ಹಲವಾರು ಮೆಟ್ಟಿಲುಗಳನ್ನು ಹತ್ತುವುದು	☐	☐	☐	☐	☐
ಕೆಳಗೆ ಕುಳಿತುಕೊಳ್ಳುವುದು/ ಮಂಡಿಯೂರುವುದು	☐	☐	☐	☐	☐
ಕೆಳಗೆ ಬಿದ್ದ ವಸ್ತುವನ್ನು ಎತ್ತಿಕೊಳ್ಳುವುದು	☐	☐	☐	☐	☐
ಯಾವುದೇ ಸಹಾಯವಿಲ್ಲದೆ ನೆಲದಿಂದ ಮೇಲೇಳುವುದು	☐	☐	☐	☐	☐
ಕಡಿಮೆ ಎತ್ತರ ಇರುವ ಕೈಗಳಿಲ್ಲದ ಅಥವಾ ಹಿಡಿಯಲು ಏನೂ ಇಲ್ಲದ ಕುರ್ಚಿಯಿಂದ ಏಳುವುದು	☐	☐	☐	☐	☐
ಸಾಮಾನ್ಯವಾಗಿ ಕುಳಿತಲ್ಲಿಂದ ಮೇಲೇಳುವುದು	☐	☐	☐	☐	☐
ಭಾರದ ವಸ್ತುವನ್ನು ಎತ್ತುವುದು(ಭಾರವಾದ ಚೀಲಗಳು, ನೀರು ತುಂಬಿದ ಭಾರದ ಪಾತ್ರೆ ಇತ್ಯಾದಿ)	☐	☐	☐	☐	☐
ಬಾಟಲಿ ಅಥವಾ ಜಾರಿನ ಮುಚ್ಚಳ ತೆರೆಯುವುದು	☐	☐	☐	☐	☐
ಸಾರ್ವಜನಿಕ ಸಾರಿಗೆಯನ್ನು ಬಳಸುವುದು	☐	☐	☐	☐	☐
ಕಾರನ್ನು ಹತ್ತುವುದು ಅಥವಾ ಇಳಿಯುವುದು	☐	☐	☐	☐	☐
ಶಾಪಿಂಗ್ (ವಸ್ತುಗಳನ್ನು ಖರೀದಿ) ಮಾಡುವುದು	☐	☐	☐	☐	☐
ಮನೆ ಕೆಲಸವನ್ನು ಮಾಡುವುದು (ಹಾಸಿಗೆಯನ್ನು ಹಾಸುವುದು ಮತ್ತು ಮಡಚುವುದು, ಗುಡಿಸುವುದು, ಒರೆಸುವುದು, ಬಟ್ಟೆಯನ್ನು ಇಸ್ತ್ರಿ ಮಾಡುವುದು, ಪಾತ್ರೆ ತೊಳೆಯುವುದು ಇತ್ಯಾದಿ)	☐	☐	☐	☐	☐

Copyright© - January 2015 - All right Reserved

18. ನಿಮ್ಮ ಸ್ನಾಯು (ಮಾಂಸಖಂಡದ) ದೌರ್ಬಲ್ಯವು ನಿಮ್ಮ ಚಲನೆಯನ್ನು ಅಡ್ಡಿಗೊಳಿಸುತ್ತಿದೆಯೇ?

- ☐ ಹೌದು, ತುಂಬಾ
- ☐ ಹೌದು, ಸ್ವಲ್ಪಮಟ್ಟಿಗೆ
- ☐ ಹೌದು, ಸ್ವಲ್ಪ
- ☐ ಇಲ್ಲವೇ ಇಲ್ಲ

19. 18 ನೇ ಪ್ರಶ್ನೆಗೆ ಉತ್ತರ "ಹೌದು" ಎಂದಾದರೆ, ಏಕೆ?
(ನೀವು ಇಷ್ಟಪಡುವಷ್ಟು ಉತ್ತರಗಳನ್ನು ಆಯ್ದುಕೊಳ್ಳಬಹುದು)

- ☐ ನೋವಿನ ಭಯ
- ☐ ನಿಮ್ಮಿಂದ ಸಾಧ್ಯವಾಗದಿರಬಹುದು ಎಂಬ ಭಯ
- ☐ ಈ ಚಟುವಳಿಕೆಗಳ ನಂತರ ದಣಿದ ಭಾವನೆಯ ಭಯ
- ☐ ಬೀಳುವ ಭಯ
- ☐ ಇತರೆ:

20. ನಿಮ್ಮ ಸ್ನಾಯುವಿನ (ಮಾಂಸಖಂಡದ) ದುರ್ಬಲತೆ ನಿಮ್ಮ ಲೈಂಗಿಕ ಜೀವನಕ್ಕೆ ಅಡ್ಡಿಯಾಗುತ್ತದೆಯೇ?

- ☐ ನಾನು ಲೈಂಗಿಕ ಆಸಕ್ತಿಯನ್ನು ಹೊಂದಿಲ್ಲ (ಲೈಂಗಿಕವಾಗಿ ಸಕ್ರಿಯವಾಗಿಲ್ಲ)
- ☐ ಹೌದು, ಸಂಪೂರ್ಣವಾಗಿ
- ☐ ಹೌದು, ಸ್ವಲ್ಪಮಟ್ಟಿಗೆ
- ☐ ಹೌದು, ಸ್ವಲ್ಪ
- ☐ ಇಲ್ಲ, ಇಲ್ಲವೇ ಇಲ್ಲ

21. ನಿಮ್ಮ ದೈಹಿಕ ಚಟುವಟಿಕೆ/ ಕ್ರೀಡೆಗಳಲ್ಲಿ ಭಾಗವಹಿಸುವಿಕೆ ಹೇಗೆ ಬದಲಾಗಿದೆ?

- ☐ ಹೆಚ್ಚಾಗಿದೆ
- ☐ ಕಡಿಮೆಯಾಗಿದೆ
- ☐ ಏನೂ ಬದಲಾಗಿಲ್ಲ
- ☐ ನಾನು ಎಂದಿಗೂ ದೈಹಿಕ ಚಟುವಟಿಕೆ / ಕ್ರೀಡೆಗಳಲ್ಲಿ ಭಾಗವಹಿಸಿಲ್ಲ

22. ನಿಮ್ಮ ಬಿಡುವಿನ (ವಿರಾಮದ) ಸಮಯದ ಚಟುವಟಿಕೆಗಳಲ್ಲಿ
(ಸಮಾರಂಭಗಳಲ್ಲಿ ಭಾಗವಹಿಸುವುದು, ತೋಟಗಾರಿಕೆ, ನಿಮಗೆ ಇಷ್ಟವಾದ ಯಾವುದೇ ಕೆಲಸವನ್ನು ಮಾಡುವುದು, ಮಕ್ಕಳೊಂದಿಗೆ ಆಟ ಆಡುವುದು, ಹಿರಿಯ ನಾಗರಿಕರ ಕ್ಲಬ್‌ಗೆ ಹೋಗುವುದು, ಕಾರ್ಡ್(ಇಸ್ಪೀಟ್) ಆಡುವುದು, ವಾಕಿಂಗ್ ಮಾಡುವುದು ಇತ್ಯಾದಿ)
ನಿಮ್ಮ ಭಾಗವಹಿಸುವಿಕೆ ಹೇಗೆ ಬದಲಾಗಿದೆ?

- ☐ ಹೆಚ್ಚಾಗಿದೆ
- ☐ ಕಡಿಮೆಯಾಗಿದೆ
- ☐ ಏನೂ ಬದಲಾಗಿಲ್ಲ
- ☐ ನಾನು ಎಂದಿಗೂ ವಿರಾಮದ ಚಟುವಟಿಕೆಗಳಲ್ಲಿ ಭಾಗವಹಿಸಿಲ್ಲ

Copyright© - January 2015 - All right Reserved
