# Supplementary material for: Psychometric performance of the Kannada version of sarcopenia quality of life questionnaire (SarQoL®)
Source: BMC Musculoskelet Disord. 2023 Jun 2;24:445. doi: 10.1186/s12891-023-06559-8 (PMC10236591; doi:10.1186/s12891-023-06559-8)
Supplement: Supplementary file 7 — Supplementary Material 7 [file 12891_2023_6559_MOESM7_ESM.pdf]

Supplementary material 7: Description of replacement of words in SarQoL®-Kannada

Words	Kannada literal meaning	Replacement
DIY (in question no. 3)	No exact word	Nimage iṣṭavāda kelasavannu māḍuvudu
Washing-up (in question no. 3)	Toḷeyuvudu-ap (Referred Hindi version)	Snāna māḍuvudu
Vacuum cleaning (in question no. 4 & 17)	nirvata suchigolivike	Vyākyum klīniṅg māḍuvudu
Arm rest (in question no. 17)	No exact word	Kaḍime ettariruva (kaigaḷu) illade iruva kurciyinda mēlēḷuvudu
Banister (in question no. 17)	No exact word (Referred Hindi version)	Byāniṣṭar (hyāṇḍ rēl)
Playing bridge (in question no. 22)	No exact word (Referred Hindi version)	Kārḍ (ispīṭ) āḍuvudu
