# Supplementary material for: Psychometric performance of the Kannada version of sarcopenia quality of life questionnaire (SarQoL®)
Source: BMC Musculoskelet Disord. 2023 Jun 2;24:445. doi: 10.1186/s12891-023-06559-8 (PMC10236591; doi:10.1186/s12891-023-06559-8)
Supplement: Supplementary file 8 — Supplementary Material 8 [file 12891_2023_6559_MOESM8_ESM.pdf]

Supplementary material 8: Description of replacement of words in SarQoL®-Kannada

Words	Initial choice	Replacement
Some (in question no. 1)	Kelavu	Svalpa maṭṭige
Light (in question no. 3)	Laghuvāda	Haguravāda
Moderate (in question no. 4)	Madhyama	Kaṣṭakaravallada
Physical appearance (in question no. 13)	Daihika rūpadalli	Dēhada ākāradalli
Squatting or Kneeling (in question no. 17; item 4)	Maṇḍiyūruvudu	Keḷage kuḷitukoḷḷuvudu/ Maṇḍiyūruvudu
Stooping or leaning down (in question no. 17; item 5)	Baggi tegeyuvudu	Ettikoḷḷuvudu
Limit your movement? (in question no. 18)	Mitagoḷisuttideyē?	Aḍḍi goḷisuttiddeyē?
Changed? (in question no.21)	Badalāvaṇeyāgide?	Badalāgide
