# Supplementary material for: Psychometric performance of the Kannada version of sarcopenia quality of life questionnaire (SarQoL®)
Source: BMC Musculoskelet Disord. 2023 Jun 2;24:445. doi: 10.1186/s12891-023-06559-8 (PMC10236591; doi:10.1186/s12891-023-06559-8)
Supplement: Supplementary file 9 — Supplementary Material 9 [file 12891_2023_6559_MOESM9_ESM.pdf]

Supplementary material 9: Description of alternative item statement used in SarQoL®-Kannada

**Words**	**Initial choice**	**Replacement**
Choose as many answers as you like (in question no. 7)	Ī keḷagina āykegaḷalli nimage iṣṭavāda ondakkinta heccina āykegaḷannu āydukoḷḷabahudu	Nīvu iṣṭapaḍuvaṣṭu uttaragaḷannu āydukoḷḷabahudu
I feel a weakness in the muscles (in question no. 7; item 3)	Nānu snāyugaḷalli daurbalyavannu hondiddēne	Nanna snāyugaḷu durbalavāgive endu bhāvisuttēne
I've had to face the death of several people close to me (in question no. 7; item 5)	Nanna hattiradavara halavāru janara sāvannu nānu edurisiruttēne	Nanna hattiradavara halavāru janara sāvannu nōḍiddēne
I do not have much energy, I am often tired (in question no. 3; item 6)	Nānu śaktihīnanāgiruttēne	Nanage heccu śakti illa
My eyesight is poor (in question no. 7; item 7)	Nānu kaḷape dr̥ṣṭiyanu hondiddēne	Nanna kaṇṇina dr̥ṣṭi mandavāgide
Do you feel physically weak? (question no.8)	Nīvu daihikavāgi daurbalyavannu anubhavisuttiddīrā?	Nīvu daihikavāgi durbalarāgiddīrā?
Do you feel you are limited in: (question no. 9)	Ivugaḷalli nīvu sīmitarendu bhāvisuttīrā?	Ī keḷagina caṭuvaṭikegaḷalli
The length of your steps (question 9; item 5)	Hejje iṭṭu	Hejje iṭṭu (Hejjegaḷa naḍuvina antara)
Choose as many answers as you like (in question no. 14)	Ī keḷaginavugaḷalli ondakkinta heccina āykegaḷannu āydukoḷḷabahudu	Ī keḷaginavugaḷalli nim'ma āykegaḷannu āydukoḷḷabahudu
Loss of height (in question no. 14; item 3)	Ettarada naṣṭavāgide	Ettara kaḍimeyāgide
Loss of muscle mass (in question no. 14; item 4)	Snāyu (mānsa khaṇḍa) vina balavannu kaḷedukoṇḍiddēne	Mānsakhaṇḍagaḷu (snāyugaḷu) kṣīṇisuttive
Hair loss (in question no. 14; item 5)	Kūdalu uduruvikeyannu hondiddēne	Kūdalu uduruttide
Getting white or grey hair (in question no. 14; item 6)	Biḷi athavā būdu baṇṇada kūdalannu hondiddēne	Kūdalu biḷiyāguttide
Choose as many answers as you like (in question no. 19)	Nimage iṣṭavādantaha ondakkinta heccina āykeyannu āydukoḷḷabahudu	Nim'ma āykegaḷannu āydukoḷḷabahudu
I am not sexually active (in question no. 20; item 1)	Nānu laiṅgika āsaktiyannu hondilla	Nānu laiṅgika āsaktiyannu hondilla (laiṅgikavāgi sakriyavāgilla)
How has your participation in leisure activities (going out to eat, gardening, doing DIY, shooting/fishing, senior citizens clubs, playing bridge, going for a walk, etc.) changed? (question no. 22)	Hēge nim'ma biḍuvina (virāmada) samayada caṭuvaṭikegaḷa bhāgavahisuvike (Tinnalu horage hōguvudu, tōṭagārike, nimage iṣṭavāda yāvudē kelasavannu māḍuvudu, śūṭiṅg/ mīnu hiḍiyuvudu, hiriya nāgarikara klab ge hōguvudu, kārḍ(isṭīṭ) āḍuvudu, vākiṅg māḍuvudu ityādi) Badalāgide?	Nim'ma biḍuvina (virāmada) samayada caṭuvaṭikegaḷalli (Tinnalu horage hōguvudu, tōṭagārike, nimage iṣṭavāda yāvudē kelasavannu māḍuvudu, śūṭiṅg/ mīnu hiḍiyuvudu, hiriya nāgarikara klabge hōguvudu, kārḍ(isṭīṭ) āḍuvudu, vākiṅg māḍuvudu ityādi) nim'ma bhāgavahisuvike hēge badalāgide?
