# Supplementary material for: Psychometric performance of the Kannada version of sarcopenia quality of life questionnaire (SarQoL®)
Source: BMC Musculoskelet Disord. 2023 Jun 2;24:445. doi: 10.1186/s12891-023-06559-8 (PMC10236591; doi:10.1186/s12891-023-06559-8)
Supplement: Supplementary file 10 — Supplementary Material 10 [file 12891_2023_6559_MOESM10_ESM.pdf]

Supplementary material 10: Feedback from the participants after testing of pre-final version

**Question number**	**Words difficult to understand**	**Number of participants**
Question no. 1	Tōḷugaḷallina, Snāyu, namyate, nim'ma snāyuvina namyate?	2
Question no. 4	Kaṣṭakaravallada, vyākyūm klīniṅg māḍuvudu, tōṭadallina kaḷeyannu	3
Question no. 6	Prastuta	2
Question no. 9	Nīvu eṣṭu bāri naḍedukoṇḍu hōgaballiri?	1
Question no. 11	Samatōlanadalli	2
Question no. 15	Asamādhānagoṇḍiddīrā	2
Question no. 17	Byānisṭar (hyāṇḍrēl), Ettariruva, (Ārms resṭ)	3
