# Supplementary material for: Psychometric performance of the Kannada version of sarcopenia quality of life questionnaire (SarQoL®)
Source: BMC Musculoskelet Disord. 2023 Jun 2;24:445. doi: 10.1186/s12891-023-06559-8 (PMC10236591; doi:10.1186/s12891-023-06559-8)
Supplement: Supplementary file 11 — Supplementary Material 11 [file 12891_2023_6559_MOESM11_ESM.pdf]

Supplementary material 11: Changes in the words/sentences after discussion of participants feedback with the experts

Muscle (in question no. 1; item 3)	Snāyu	Added Mānsakhaṇḍa in brackets
In your arms (in question no. 1; item 1)	Tōḷugaḷallina	Kaigaḷallina
Your muscle flexibility (in question no. 1; item 6)	Nim'ma snāyuvina namyate?	Nim'ma snāyuvina (mānsakhaṇḍada) namyate? [Nim'ma mānsa khaṇḍagaḷu sulabhavāgi dēhavannu baggisalu mattu kaikālugaḷannu maḍacalu mattu cācalu sahakarisuttiveye?]
Vacuum cleaning (in question no. 4)	Vyākyūm klīniṅg	Guḍisuvudu, oresuvudu
Moderate (in question no.4)	Kaṣṭakaravallada	Tumbā kaṣṭavallada
Pulling up weed in the garden (in question no. 4)	Tōṭadallina kaḷeyannu tegeyuvudu	Tōṭadallina hullu athavā bēḍavāda giḍagaḷannu tegeyuvudu
Currently (in question no. 6)	Prastuta	Īga
Do you have problems with your balance? (question no. 11)	Nim'ma samatōlanadalli samasyeyannu hondiruvirā?	Nim'ma samatōlanadalli (byālens – nēravāgi nilluvāga athavā naḍeyuvāga om'mege bīḷuvante āguttadeyē?) Samasyeyannu hondiruvirā?
Going up one or several steps without holding on to the banister (in question no. 17; item 3)	Byānisṭar (hyāṇḍ rēl) annu hiḍidukoḷḷade ondu athavā halavāru mahaḍiya meṭṭilannu hattuvudu	Meṭṭilu hattuvāga gōḍe athavā bēre yāvudara sahāyavillade ondu athavā halavāru meṭṭilugaḷannu hattuvudu
Getting out of a low chair without arm rests (in question no. 17; item 7)	Kaḍime ettaraviruva kaigaḷillade (ārm resṭ) iruva kurciyinda mēlēḷuvudu	Kaḍime ettara iruva kaigaḷillada athavā hiḍiyalu ēnū illada kurciyinda ēḷuvudu
How has your participation in leisure activities (going out to eat, gardening, doing DIY, shooting/fishing, senior citizens clubs, playing bridge, going for a walk, etc.) changed? (question no. 22)	Nim'ma biḍuvina (virāmada) samayada caṭuvaṭikegaḷalli (tinnalu horage hōguvudu, tōṭagārike, nimage iṣṭavāda yāvudē kelasavannu māḍuvudu, śūṭiṅg/ mīnu hiḍiyuvudu, hiriya nāgarikara klabge hōguvudu, kārḍ(isṭīṭ) āḍuvudu, vākiṅg māḍuvudu ityādi) nim'ma bhāgavahisuvike hēge badalāgide?	Nim'ma biḍuvina (virāmada) samayada caṭuvaṭikegaḷalli (samārambhagaḷalli bhāgavahisuvudu, tōṭagārike, nimage iṣṭavāda yāvudē kelasavannu māḍuvudu, makkaḷondige āṭa āḍuvudu, hiriya nāgarikara klabge hōguvudu, kārḍ(isṭīṭ) āḍuvudu, vākiṅg māḍuvudu ityādi) nim'ma bhāgavahisuvike hēge badalāgide?
