# Supplementary material for: Psychometric performance of the Kannada version of sarcopenia quality of life questionnaire (SarQoL®)
Source: BMC Musculoskelet Disord. 2023 Jun 2;24:445. doi: 10.1186/s12891-023-06559-8 (PMC10236591; doi:10.1186/s12891-023-06559-8)
Supplement: Supplementary file 12 — Supplementary Material 12 [file 12891_2023_6559_MOESM12_ESM.pdf]

Supplementary material 12: Summarization of the results of all the phases of translation and cross-cultural adaptation

Q. No.	Original	Phase 1		Phase 2	Phase 3		Phase 4	Phase 5	Final version
		**Translator 1**	**Translator 2**	**Version 1**	**Translator 3**	**Translator 4**	**Pre-test version**	**Tested**	
1	Do you currently feel you have a reduction in:<br>The strength in your arms?<br>The strength in your legs?<br>Your muscle mass?<br>Your energy?<br>Your physical capabilities?<br>Your general flexibility?	Nimage ī keḷaginavugaḷalli yāvudu modaliginta kaḍime anisuttide:<br>Kaigaḷallina śakti?<br>Kālugaḷa śakti?<br>Snāyu (mānsa khaṇḍa) gaḷa śakti?<br>Nim'ma śakti?<br>Nim'ma daihika sāmarthya?<br>Nim'ma snāyuvina namyate? (Phleksibiliṭi)	Prastuta nimage ī keḷaginavugaḷalli yāvudādaru kaḍimeyāgideyē endu anisuttadeyē?<br>Nim'ma tōḷugaḷallina śakti?<br>Nim'ma kālugaḷa śakti?<br>Snāyuśakti<br>nim'ma sāmarthya?<br>Nim'ma daihika sāmarthyagaḷu?<br>Nim'ma snāyuvina namyate?	Prastuta nimage ī keḷaginavugaḷalli yāvudādarū kaḍimeyāgideyē e0du anisuttideyē?<br>Nim'ma tōḷugaḷallina śakti?<br>Nim'ma kālugaḷa śakti?<br>Snāyu śakti?<br>Nim'ma sāmarthya?<br>Nim'ma daihika sāmarthyagaḷu?<br>Nim'ma snāyuvina namyate?	Do you feel there is decrease in any of the following?<br>Strength of your arms?<br>Strength of your legs?<br>Muscle strength?<br>Your strength?<br>Your physical abilities?<br>Your muscle flexibility?	1. At present, do you feel any of the following has lessened?<br>Strength of the arms?<br>Strength of the limbs?<br>Power of muscles?<br>Your Capability?<br>Physical Stamina?<br>Flexibility of muscles?	Prastuta nimage ī keḷaginavugaḷalli yāvudādarū kaḍimeyāgideyē e0du anisuttideyē?<br>Nim'ma tōḷugaḷallina śakti?<br>Nim'ma kālugaḷa śakti?<br>Snāyu śakti?<br>Nim'ma sāmarthya?<br>Nim'ma daihika sāmarthyagaḷu?<br>Nim'ma snāyuvina namyate?	Snāyu<br>Namyate<br>Nim'ma snāyuvina namyate?	Prastuta nimage ī keḷaginavugaḷalli yāvudādarū kaḍimeyāgideyē e0du anisuttideyē?<br>Nim'ma kaigaḷallina śakti?<br>Nim'ma kālugaḷa śakti?<br>Snāyu (mānsakhaṇḍagaḷa) śakti?<br>Nim'ma sāmarthya?<br>Nim'ma daihika sāmarthyagaḷu?<br>Nim'ma snāyuvina (mānsakhaṇḍada) namyate?<br>[Nim'ma mānsa khaṇḍagaḷu sulabhavāgi dēhavannu baggisalu mattu kaikālugaḷannu maḍacalu mattu cācalu sahakarisuttiveye?]
2	Do you have pain in your muscles?<br>Often<br>Sometimes<br>Rarely<br>Never	Nimage snāyu (mānsakhaṇḍa) gaḷalli nōvideyē?<br>Āgāga<br>kelavom'me<br>aparūpakke (viraḷa)<br>endigū illa	Nim'ma snāyugaḷalli nōvu ideyē?<br>Āgāgge<br>kelavom'me<br>viraḷavāgi<br>endigū	Nim'ma snāyugaḷalli nōvu ideyē?<br>Āgāga<br>kelavom'me<br>viraḷavāgi (aparūpakke)<br>endigū illa	Do you have pain in your muscle?<br>Most of the time<br>Sometimes<br>Rarely<br>Not at all	How often do you feel pain in the muscles?<br>Often<br>Sometimes<br>Rarely<br>Never	Nim'ma snāyugaḷalli nōvu ideyē?<br>Āgāga<br>kelavom'me<br>viraḷavāgi (aparūpakke)<br>endigū illa		Nim'ma snāyu (mānsakhaṇḍa) gaḷalli nōvu ideyē?<br>Āgāga<br>kelavom'me<br>viraḷavāgi (aparūpakke)<br>endigū illa
3	When undertaking light physical activities (walking slowly, doing the ironing, dusting, washing-up, DIY, watering the garden, etc.), do you:<br>Have difficulty?<br>Get tired?<br>Experience pain?	Nīvu laghuvāda daihika caṭuvaṭikegaḷannu māḍuvāga (nidhānavāgi naḍeyuvudu, istriyannu māḍuvudu, dhūḷannu tegeyuvudu, snāna māḍuvudu, nimage iṣṭavāda kelasavannu māḍuvudu, giḍagaḷige nīru hākuvudu ityādi.) Ivugaḷalli nimage yāvāga<br>Kaṣṭāvāguttade?<br>Sustāguttade? (Daṇivu) nōvina anubhavavāguttade?	Saṇṇa daihika caṭuvaṭikegaḷannu kaigoḷḷuvāga (nidhānavāgi naḍeyuvudu, istri māḍuvudu, dhūḷu tegeyuvudu, toḷeyuvudu, tōṭakke nīru hākuvudu, ityādi) nimage:<br>Kaṣṭavāguttadeyē?<br>Sustāguvudē?<br>Nōvāguttadeyē?	Nīvu laghuvāda daihika caṭuvaṭikegaḷannu māḍuvāga (nidhānavāgi naḍeyuvudu, istriyannu māḍuvudu, dhūḷannu tegeyuvudu, snāna māḍuvudu, nimage iṣṭavāda kelasavannu māḍuvudu, giḍagaḷige nīru hākuvudu ityādi.) Ivugaḷalli nimage yāvāga<br>Kaṣṭavāguttade?<br>Sustāguttade?<br>Nōvina anubhavavāguttade?	When you involve yourself in the lighter activities (like slow walking, ironing clothes, dusting, bathing, doing your favourite activity, watering the plants etc.) Do you feel<br>Difficult?<br>Tired?<br>Pain?	Whenever you are involved in light physical activities, (a slow walk, Ironing, Taking bath, Watering the Garden, some other activity you like) when do you feel<br>Difficult to continue?<br>Tired?<br>Pain?	Nīvu haguravāda daihika caṭuvaṭikegaḷannu māḍuvāga (nidhānavāgi naḍeyuvudu, istriyannu māḍuvudu, dhūḷannu tegeyuvudu, snāna māḍuvudu, nimage iṣṭavāda kelasavannu māḍuvudu, giḍagaḷige nīru hākuvudu ityādi.)<br>Nimage yāvāga<br>Kaṣṭavāguttade?<br>Sustāguttade?<br>Nōvina anubhavavāguttade?		Nīvu haguravāda daihika caṭuvaṭikegaḷannu māḍuvāga (nidhānavāgi naḍeyuvudu, istriyannu māḍuvudu, dhūḷannu tegeyuvudu, snāna māḍuvudu, nimage iṣṭavāda kelasavannu māḍuvudu, giḍagaḷige nīru hākuvudu ityādi.) Nimage yāvāga<br>Kaṣṭavāguttade?<br>Sustāguttade?<br>Nōvina anubhavavāguttade?

**4**	When undertaking moderate physical activities (fast walking, cleaning windows,<br>hoovering, washing the car, pulling up weeds in the garden, etc.), do you:<br>Have difficulty?<br>Get tired?<br>Experience pain?	Nīvu madhyama caṭuvaṭikegaḷannu māḍuvāga [vēgavāgi naḍeyuvudu, kiṭikigaḷannu svacchagoḷisuvudu, vyākyūm klīniṅg māḍuvudu, kārannu toḷeyuvudu, tōṭadallina kaḷeyannu tegeyuvudu (kīḷuvudu)] ivugaḷalli nimage yāvāga<br>Kaṣṭavāguttade?<br>Sustāguttade? (Daṇivu) nōvina anubhavavāguttade?	Madhyama daihika caṭuvaṭikegaḷannu kaigoḷḷuvāga (vēgada naḍige, kiṭakigaḷannu svacchagoḷisuvudu, kārannu toḷeyuvudu, tōṭadalli kaḷegaḷannu tegeyuvudu, ityādi) nimage:<br>Kaṣṭavāguttadeyē?<br>Sustāguvudē?<br>Nōvāguttadeyē?	Nīvu madhyama caṭuvaṭikegaḷannu māḍuvāga [vēgavāgi naḍeyuvudu, kiṭikigaḷannu svacchagoḷisuvudu, vyākyūm klīniṅg māḍuvudu, kārannu toḷeyuvudu, tōṭadallina kaḷeyannu tegeyuvudu (kīḷuvudu)] ivugaḷalli nimage yāvāga<br>Kaṣṭavāguttade?<br>Sustāguttade?<br>Nōvina anubhavavāguttade?	When you do a bit of heavy work such as brisk walk, cleaning windows, using vaccum cleaner, washing cars, weeding etc. Do you feel<br>It is difficult?<br>Tired?<br>Pain?	Whenever you are involved in physical activities of average physical exertion, (such as walking briskly, cleaning windows, Vacuum cleaning, washing your car, removing weeds in the farm) when do you feel<br>Difficult to continue?<br>Tired?<br>Feel pain?	Nīvu kaṣṭakaravallada caṭuvaṭikegaḷannu māḍuvāga [vēgavāgi naḍeyuvudu, kiṭikigaḷannu svacchagoḷisuvudu, vyākyūm klīniṅg māḍuvudu, kārannu toḷeyuvudu, tōṭadallina kaḷeyannu tegeyuvudu] nimage yāvāga<br>Kaṣṭavāguttade?<br>Sustāguttade?<br>Nōvina anubhavavāguttade?	Kaṣṭakarav allada Vyākyūm klīniṅg māḍuvudu Tōṭadallina kaḷeyannu	Nīvu tumbā kaṣṭavallada caṭuvaṭikegaḷannu māḍuvāga [vēgavāgi naḍeyuvudu, kiṭikigaḷannu svacchagoḷisuvudu, guḍisuvudu, oresuvudu, kārannu toḷeyuvudu, tōṭadallina hullu athavā bēḍavāda giḍagaḷannu tegeyuvudu ityādi] nimage yāvāga<br>Kaṣṭavāguttade?<br>Sustāguttade?<br>Nōvina anubhavavāguttade?
**5**	When undertaking intense physical activities (running, hiking, lifting heavy objects,<br>moving furniture, digging the garden, etc.), do you:<br>Have difficulty?<br>Get tired?<br>Experience pain?	Nīvu tīvravāda daihika caṭuvaṭikegaḷannu māḍuvāga (ōḍuvudu, tumbā dūra naḍeyuvudu, bhāravāda vastugaḷannu ettuvudu, pīṭhōpakaraṇagaḷannu calisuvudu, tōṭagaḷannu ageyuvudu ityādi) ivugaḷalli nimage yāvāga<br>Kaṣṭavāguttade?<br>Sustāguttade? (Daṇivu) nōvina anubhavavāguttade?	Tīvravāda daihika caṭuvaṭikegaḷannu kaigoḷḷuvāga (tirugāḍuvudu, bhāravāda vastugaḷannu ettuvudu, pīṭhōpakaraṇagaḷannu sarisuvudu, udyānavannu ageyuvudu, ityādi) nimage:<br>Kaṣṭavāguttadeyē?<br>Sustāguvudē?<br>Nōvāguttadeyē?	Nīvu tīvravāda daihika caṭuvaṭikegaḷannu māḍuvāga (ōḍuvudu, tumbā dūra naḍeyuvudu, bhāravāda vastugaḷannu ettuvudu, pīṭhōpakaraṇagaḷannu sarisuvudu, tōṭagaḷannu ageyuvudu ityādi) ivugaḷalli nimage yāvāga<br>Kaṣṭavāguttade?<br>Sustāguttade?<br>Nōvina anubhavavāguttade?	When you are involved in tougher activities like running, long walk, lifting heavy things, moving furniture, digging etc. Do you feel<br>It is difficult?<br>Tired?<br>Pain?	Whenever you are involved in hard physical activities, (Running, Walking a long distance, Lifting heavy objects, Digging in the farm, moving the furniture) when do you feel<br>Difficult to continue?<br>Tired?<br>Feel pain?	Nīvu tīvravāda daihika caṭuvaṭikegaḷannu māḍuvāga (ōḍuvudu, tumbā dūra naḍeyuvudu, bhāravāda vastugaḷannu ettuvudu, pīṭhōpakaraṇagaḷannu sarisuvudu, tōṭagaḷannu ageyuvudu ityādi) nimage yāvāga<br>Kaṣṭavāguttade?<br>Sustāguttade?<br>Nōvina anubhavavāguttade?		Nīvu tīvravāda daihika caṭuvaṭikegaḷannu māḍuvāga (ōḍuvudu, tumbā dūra naḍeyuvudu, bhāravāda vastugaḷannu ettuvudu, pīṭhōpakaraṇagaḷannu sarisuvudu, tōṭagaḷannu ageyuvudu ityādi) nimage yāvāga<br>Kaṣṭavāguttade?<br>Sustāguttade?<br>Nōvina anubhavavāguttade?
**6**	Do you currently feel old?<br>Yes, very<br>Yes, somewhat<br>Yes, a little<br>No, not at all	Nīvu nim'mannu vr̥d'dha athavā vr̥d'dhe endu bhāvisuvirā?<br>Haudu, tumbā<br>haudu, svalpamaṭṭige<br>haudu, svalpa<br>illa, illavē illa	Nimage prastuta vayas'sāguttide endu anisuttideyē?<br>Haudu, tumbā<br>haudu, svalpamaṭṭige<br>haudu svalpa<br>illa, illavē illa	Prastuta nimage vayas'sāguttide endu bhāvisuvirā?<br>Haudu, tumbā<br>haudu, svalpamaṭṭige<br>haudu, svalpa<br>illa, illavē illa	Do you feel that you are getting old?<br>Yes, too often<br>Yes, sometimes<br>Yes, a little<br>No, not at all	Do you think that you are getting aged now?<br>Yes, very much<br>Yes, to some extent<br>Yes, a little<br>No, Never	Prastuta nimage vayas'sāguttide endu bhāvisuvirā?<br><br>Haudu, tumbā<br>haudu, svalpamaṭṭige<br>haudu, svalpa<br>illa, illavē illa	Prastuta	Īga nimage vayas'sāguttide endu bhāvisuvirā?<br>Haudu, tumbā<br>haudu, svalpamaṭṭige<br>haudu, svalpa<br>illa, illavē illa
**7**	If yes to question 6, what gives you that impression?	Āranē saṅkhyeya praśnege uttara"haudu" endādare, nimage hāge ēke	6 Nē praśnege haudu endādare, nimage ēke hāge anisitu? (Nimage	6 Nē praśnege uttara"haudu" endādare, nimage hāge ēke anisuttide? (Ī keḷagina	If your reply is yes for Q6. Why do you feel so?	If your answer for the sixth question is 'yes',	6 Nē praśnege uttara"haudu" endādare, nimage hāge ēke		6 Nē praśnege uttara"haudu" endādare, nimage hāge ēke anisuttide? (Nīvu iṣṭapaḍuvaṣṭu

	(Choose as many answers as you like)<br>I become unwell easily<br>I take many medications<br>I feel a weakness in my muscles<br>I have problems with my memory<br>I've had to face the death of several people close to me<br>I do not have much energy, I am often tired<br>My eyesight is poor<br>Other:	anisuttide? (Ī keḷagina āykegaḷalli nimage iṣṭavāda ondakkinta heccina āykegaḷannu āydakoḷḷi)<br>Nānu sulabhavāgi anārōgyakke tuttāguttēne<br>nānu anēka auṣadhagaḷannu tegedukoḷḷutēne.<br>Nānu snāyugaḷalli daurbalyavannu anubhavisuttiddēne.<br>Nānu marevina samasyeyannu hondiddēne.<br>Nanna hattiradavara halavāru janara sāvannu nānu edurisiruttēne<br>nānu śaktihīnanāgiruttēne,nanage āgāga sustāguttade.<br>Nānu kaḍime kaṇṇina dr̥ṣṭiyannu hondiddēne.<br>Itare.	bēkāda uttaragaḷannu āykemāḍi)<br>Nānu sulabhavāgi asvasthanāguttēne<br>nānu anēka auṣadhigaḷannu tegedukoḷḷuttēne<br>nānu snāyugaḷalli daurbalyavannu anubhavisuttēne<br>nanage nenapina śaktiya samasyeyide<br>nanna hattiraviruva halavāru janara sāvannu nānu edurisabēkāyitu<br>nanage heccu śakti illa, nānu āgāgge daṇididdēne<br>kaḷape dr̥ṣṭi<br>itare	āykegaḷalli nimage iṣṭavāda ondakkinta heccina āykegaḷannu āydukoḷḷabahudu)<br>Nānu sulabhavāgi anārōgyakke tuttāguttēne<br>nānu anēka auṣadhagaḷannu tegedukoḷḷutēne.<br>Nānu snāyugaḷalli daurbalyavannu anubhavisuttiddēne.<br>Nānu marevina samasyeyannu hondiddēne.<br>Nanna hattirada halavāru janara sāvannu nānu edurisiruttene.<br>Nānu śaktihīnanāgiruttēne, nanage āgāga sustāguttade.<br>Nānu kaḷape dr̥ṣṭiyannu hondiddēne.<br>Itare.	(Choose one or more than one from the options given below)<br>I feel sick easily<br>I am taking a lot of medicine<br>I feel weakness in the muscles<br>I have memory issues<br>I have lost my near dear ones<br>I am weak and get tired often<br>I have poor eyesight<br>Others	why do you think so? (You can choose more than one of the following options if you prefer to)<br>I Fall sick very often<br>I take too much medication<br>I feel my muscles are weak<br>I forget things very easily<br>I have confronted with deaths of closer ones<br>I feel week; get tired very easily<br>I have poor vision<br>Other reasons	anisuttide? (Nīvu iṣṭapaḍuvaṣṭu uttaragaḷannu āydukoḷḷabahudu)<br>Nānu sulabhavāgi anārōgyakke tuttāguttēne<br>nānu anēka auṣadhagaḷannu tegedukoḷḷutēne.<br>Nanna snāyugaḷu durbalavāgive endu bhāvisuttēne<br>nānu marevina samasyeyannu hondiddēne.<br>Nanna hattiradavara halavāru janara sāvannu nōḍiddēne<br>nanage heccu śakti illa, nanage āgāga sustāguttade.<br>Nanna kaṇṇina dr̥ṣṭi mandavāgide<br>itare.		uttaragaḷannu āydukoḷḷabahudu)<br>Nānu sulabhavāgi anārōgyakke tuttāguttēne<br>nānu anēka auṣadhagaḷannu tegedukoḷḷutēne.<br>Nanna snāyugaḷu (mānsakhaṇḍa) durbalavāgive endu bhāvisuttēne<br>nānu marevina samasyeyannu hondiddēne.<br>Nanna hattiradavara halavāru janara sāvannu nōḍiddēne<br>nanage heccu śakti illa, nanage āgāga sustāguttade.<br>Nanna kaṇṇina dr̥ṣṭi mandavāgide<br>itare.
**8**	Do you feel physically weak?<br>Yes, completely<br>Yes, somewhat<br>Yes, a little<br>No, not at all	Nīvu daihikavāgi daurbalyavannu anubhavisuttiddīrā?<br>Haudu, sampūrṇavāgi<br>haudu, svalpamaṭṭige<br>haudu, svalpa<br>illa, illavē illa	Nīvu daihikavāgi durbalarāgiddīrā?<br>Haudu, sampūrṇavāgi<br>haudu, svalpamaṭṭige<br>haudu svalpa<br>illa, illavē illa	Nīvu daihikavāgi daurbalyavannu anubhavisuttiddīrā?<br>Haudu, sampūrṇavāgi<br>haudu, svalpamaṭṭige<br>haudu, svalpa<br>illa, illavē illa	Do you feel physically weak?<br>Most of the time<br>Sometimes<br>Rarely<br>Not at all	Do you experience physical weakness?<br>Yes, completely<br>Yes, to some extent<br>Yes, a little<br>No, Never.	Nīvu daihikavāgi durbalarāgiddīrā?<br>Haudu, sampūrṇavāgi<br>haudu, svalpamaṭṭige<br>haudu, svalpa<br>illa, illavē illa		Nīvu daihikavāgi durbalarāgiddīrā?<br>Haudu, sampūrṇavāgi<br>haudu, svalpamaṭṭige<br>haudu, svalpa<br>illa, illavē illa
**9**	Do you feel you are limited in:<br>The length of time you can walk for?<br>How often you go out walking?<br>The distance you can walk?<br>The speed at which you can walk?<br>The length of your steps?	Ivugaḷalli nīvu sīmitarendu bhāvisuttīrā?<br>Nīvu ondu nigadita samayakkinta heccu naḍeyalu sādhyavāgadiruvudu<br>nīvu eṣṭu bāri vākiṅg ge hōgaballiri?<br>Nīvu eṣṭu dūra naḍeyaballiri?<br>Nīvu eṣṭu vēgavāgi naḍeyaballiri?<br>Nīvu eṣṭu dūra hejje iṭṭu naḍeyaballiri?	Nīvu ī keḷaginavugaḷige sīmitavāgiruttīri endu bhāvisuttīrā?<br>Nīvu eṣṭu samayadavarege naḍeyabahudu?<br>Nīvu eṣṭu bāri naḍedukoṇḍu hōguttīri?<br>Nīvu naḍeyabahudāda dūra?<br>Nīvu naḍeyuva vēga?<br>Nim'ma hejjegaḷa udda?	Ivugaḷalli nīvu sīmitarendu bhāvisuttīrā?<br>Nīvu eṣṭu samaya naḍeyaballiri?<br>Nīvu eṣṭu bāri naḍedukoṇḍu hōgaballiri?<br>Nīvu eṣṭu dūra naḍeyaballiri?<br>Nīvu eṣṭu vēgavāgi naḍeyaballiri?<br>Nīvu eṣṭu dūra hejje iṭṭu naḍeyaballiri?	What do you think about the following activities?<br>How long can you walk?<br>How many times can you walk?<br>How far can you walk?<br>How fast can you walk?<br>How much distance can you	Do you think you are limited in the following activities?<br>How long (time) can you walk?<br>How many times can you walk<br>How far can you walk?<br>How fast can you walk?<br>How much distance can you	Ī keḷagina caṭuvaṭikegaḷalli,<br>Nīvu eṣṭu samaya naḍeyaballiri?<br>Nīvu eṣṭu bāri naḍedukoṇḍu hōgaballiri?<br>Nīvu eṣṭu dūra naḍeyaballiri?<br>Nīvu eṣṭu vēgavāgi naḍeyaballiri?<br>Nīvu eṣṭu dūra hejje iṭṭu (hejjegaḷa naḍuvina antara) naḍeyaballiri?	Nīvu eṣṭu bāri naḍedukoṇḍu hōgaballiri?	Ī keḷagina yāva caṭuvaṭike kaḍimeyāgide (sīmita) endu bhāvisuttīri:<br>Nīvu eṣṭu samaya naḍeyaballiri?<br>Nīvu eṣṭu bāri naḍedukoṇḍu hōgaballiri?<br>Nīvu eṣṭu dūra naḍeyaballiri?<br>Nīvu eṣṭu vēgavāgi naḍeyaballiri?<br>Nīvu eṣṭu dūra hejje iṭṭu (hejjegaḷa naḍuvina antara) naḍeyaballiri?

					keep between steps?	keep between steps?			
**10**	When you are walking:<br>Do you feel very tired?<br>Do you need to sit down regularly to recover?<br>Do you have difficulty crossing roads quickly enough?<br>Do you have difficulties with uneven surfaces?	Nīvu naḍeyuvāga (vākiṅg māḍuvāga):<br>Nimage tumbā sustu (daṇivu) anubhavavāguttadeyē?<br>Cētarisikoḷḷalu nīvu niyamitavāgi kuḷitukoḷḷabēkāgattadeyē?<br>Nīvu rastegaḷannu vēgavāgi dāṭalu kaṣṭapaḍuttīrā?<br>Nīvu asama mēlmaigaḷalli (samataṭṭallada jāgadalli) naḍeyalu tondareyannu hondiruvirā?	Nīvu naḍeyuvāga<br>Nimage tumbā āyāsavāguttadeye?<br>Cētarisikoḷḷalu nīvu āgāga kuḷitukoḷḷabēkē?<br>Nimage rastegaḷannu tvaritavāgi dāṭalu kaṣṭavideyē?<br>Asama mēlmaigaḷondige nīvu tondaregaḷannu hondiddīrā?	Nīvu naḍeyuvāga (vākiṅg māḍuvāga):<br>Nimage tumbā āyāsada anubhavavāguttadeyē?<br>Cētarisikoḷḷalu nīvu āgāga kuḷitukoḷḷabēkāgattadeyē?<br>Nīvu rastegaḷannu vēgavāgi dāṭalu kaṣṭapaḍuttīrā?<br>Nīvu samataṭṭallada jāgadalli naḍeyalu tondareyannu hondiruvirā?	While walking:<br>Do you feel tired?<br>Do you sit in between to feel comfortable?<br>Do you find it difficult to cross the road quickly?<br>It is difficult to walk on plain surface?	When you walk/ go on walking:<br>Do you feel tired?<br>Do you need to sit for some time while working in order to regain stamina?<br>Do you find it hard to cross the road very fast?<br>Do you have trouble in walking on uneven space?	Nīvu naḍeyuvāga (vākiṅg māḍuvāga):<br>Nimage tumbā āyāsada anubhavavāguttadeyē?<br>Cētarisikoḷḷalu nīvu āgāga kuḷitukoḷḷabēkāgattadeyē?<br>Nīvu rastegaḷannu vēgavāgi dāṭalu kaṣṭapaḍuttīrā?<br>Nīvu samataṭṭallada jāgadalli naḍeyalu tondareyannu hondiruvirā?		Nīvu naḍeyuvāga (vākiṅg māḍuvāga):<br>Nimage tumbā āyāsada anubhavavāguttadeyē?<br>Cētarisikoḷḷalu nīvu āgāga kuḷitukoḷḷabēkāgattadeyē?<br>Nīvu rastegaḷannu vēgavāgi dāṭalu kaṣṭapaḍuttīrā?<br>Nīvu samataṭṭallada jāgadalli naḍeyalu tondareyannu hondiruvirā?
**11**	Do you have problems with your balance?<br>Often<br>Occasionally<br>Rarely<br>Never	Nim'ma samatōlanadalli samasyeyannu hondiruvirā?<br>Āgāga<br>om'mom'me<br>aparūpakke<br>endigū illa	Nim'ma samatōlanadalli nimage samasye ideyē?<br>Āgāgge<br>kelavom'me<br>viraḷavāgi<br>endigū	Nim'ma samatōlanadalli samasyeyannu hondiruvirā?<br>Āgāga<br>om'mom'me<br>aparūpakke<br>endigū illa	Do you have problem in maintaining stability?<br>Most of the time<br>Sometimes<br>Rarely<br>Not at all	Do you feel that that you lose your balance ?<br>Often<br>Sometimes<br>Rarely<br>Never	Nim'ma samatōlanadalli samasyeyannu hondiruvirā?<br>Āgāga<br>om'mom'me<br>aparūpakke<br>endigū illa	Samatōlana dalli	Nim'ma samatōlanadalli (byālens – nēravāgi nilluvāga athavā naḍeyuvāga om'mege bīḷuvante āguttadeyē?)<br>Samasyeyannu hondiruvirā?<br>Āgāga<br>om'mom'me<br>aparūpakke<br>endigū illa
**12**	How often do you fall?<br>Very often<br>Occasionally<br>Rarely<br>Never	Nīvu eṣṭu bāri biddidīri?<br>Bahaḷaṣṭu sala<br>om'mom'me<br>aparūpakke<br>endigū illa	Nīvu eṣṭu bāri bīḷuttīri?<br>Āgāgge<br>kelavom'me<br>viraḷavāgi<br>endigū	Nīvu eṣṭu bāri bīḷuttīri?<br>Bahaḷaṣṭu sala<br>om'mom'me<br>aparūpakke<br>endigū illa	How many times do you stumble?<br>Most of the time<br>Sometimes<br>Rarely<br>Not at all	How many times do you fall losing your balance?<br>Many times<br>Sometimes<br>Rarely<br>Never	Nīvu eṣṭu bāri bīḷuttīri?<br>Bahaḷaṣṭu sala<br>om'mom'me<br>aparūpakke<br>endigū illa		Nīvu eṣṭu bāri bīḷuttīri?<br>Bahaḷaṣṭu sala<br>om'mom'me<br>aparūpakke<br>endigū illa
**13**	Do you think that your physical appearance has changed?<br>Yes, very<br>Yes, somewhat<br>Yes, a little<br>No, not at all	Nīvu nim'ma daihika rūpadalli badalāvaṇeyāgide endu bhāvisuttīrā?<br>Haudu, tumbā<br>haudu, svalpamaṭṭige<br>haudu, svalpa<br>illa. Illavē illa	Nim'ma daihika nōṭa badalāgide endu nīvu bhāvisuttīrā?<br>Haudu, tumbā<br>haudu, svalpamaṭṭige<br>haudu svalpa<br>illa, illavē illa	Nīvu nim'ma daihika rūpadalli badalāvaṇeyāgide endu bhāvisuttīrā?<br>Haudu, tumbā<br>haudu, svalpamaṭṭige<br>haudu, svalpa<br>illa. Illavē illa	Do you feel there is change in your physical appearance?<br>Yes, a lot<br>Yes, to some extent<br>Yes, a little<br>No, not at all	Do you think that there is a change in the physical structure of your body?<br>Yes, to a great extent<br>Yes, to some extent<br>Yes, a little<br>No, Never	Nīvu nim'ma dēhada ākāradalli badalāvaṇeyāgide endu bhāvisuttīrā?<br>Haudu, tumbā<br>haudu, svalpamaṭṭige<br>haudu, svalpa<br>illa. Illavē illa		Nīvu nim'ma dēhada ākāradalli badalāvaṇeyāgide endu bhāvisuttīrā?<br>Haudu, tumbā<br>haudu, svalpamaṭṭige<br>haudu, svalpa<br>illa. Illavē illa

**14**	If yes to question 13, in what way? (Choose as many answers as you like)<br>Change in your weight (you've put on weight or you've lost weight)<br>Appearance of wrinkles<br>Loss of height<br>Loss of muscle mass<br>Hair loss<br>Getting white or grey hair<br>Other:	Hadimūranē (13 nē) saṅkhyeya praśnege uttara"haudu" endādare, adu yāva rītiyalli? (Ī keḷaginavugaḷalli ondakkinta heccu āykegaḷannu āydukoḷḷabahudu)<br>Nim'ma tūkadallina badalāvaṇe(nīvu tūkavannu heccisiddīri/ nīvu tūkavannu kaḷedakoṇḍiddīri)<br>nim'ma carmada sukkugaṭṭuvike<br>ettarada naṣṭavāgide<br>snāyu (mānsa khaṇḍa) vina balavannu kaḷedukoṇḍiddēne.<br>Kūdalu uduruvikeyannu hondiddēne.<br>Biḷi athavā būdu baṇṇada kūdalannu hondiddēne<br>itare.	13Nē praśnege haudādare, yāva rītiyalli? (Nimage bēkāda uttaragaḷannu āykemāḍi)<br>Nim'ma tūkadalli badalāvaṇe (nīvu tūkavannu heccisikoṇḍiddīri athavā nīvu tūkavannu kaḷedukoṇḍiddīri)<br>sukku gōcarisuvudu<br><br>ettarada naṣṭa<br><br>snāyuśakti<br><br>kūdalu uduruvike<br><br>biḷi athavā būdu kūdalu gōcarisuvudu<br>itare	13 Nē praśnege uttara"haudu" endādare, adu yāva rītiyalli? (Ī keḷaginavugaḷalli ondakkinta heccu āykegaḷannu āydukoḷḷabahudu)<br>Nim'ma tūkadallina badalāvaṇe(nīvu tūkavannu heccisiddīri/ nīvu tūkavannu kaḷedakoṇḍiddīri)<br>nim'ma carmada sukkugaṭṭavike<br>ettarada naṣṭavāgide<br>snāyu (mānsakhaṇḍa) vina balavannu kaḷedukoṇḍiddēne<br>kūdalu uduruvikeyannu hondiddēne<br>biḷi athavā būdu baṇṇada kūdalannu hondiddēne<br>itare.	If your answer is yes to question 13, In what way do you feel there is a change<br>Difference in your body weight<br>Wrinkles on your skin<br>Have you shrunk<br>Loss of muscle strength<br>Have hair fall<br>Have gray/white hair<br>Other	If your answer to the 13th question is 'yes', in what way the change has happened? (You can choose more than one of the following options)<br>Change in your weight(You have gained/ lost weight)<br>Wrinkles on the skin<br>Lost height<br>Feel that muscles have lost power<br>Loss of hair<br>Developed White or grey hair<br>Any other change	13 Nē praśnege uttara"haudu" endādare, adu yāva rītiyalli? (Ī keḷaginavugaḷalli nim'ma āykegaḷannu āydukoḷḷabahudu)<br>Nim'ma tūkadallina badalāvaṇe(nīvu tūkavannu heccisiddīri/ nīvu tūkavannu kaḷedakoṇḍiddīri)<br>nim'ma carmada sukkugaṭṭavike<br>ettara kaḍimeyāgide mānsakhaṇḍagaḷu (snāyugaḷu) kṣīṇisuttive.<br>Kūdalu uduruttide<br>kūdalu biḷiyāguttide.<br>Itare.		13 Nē praśnege uttara"haudu" endādare, adu yāva rītiyalli? (Ī keḷaginavugaḷalli nim'ma āykegaḷannu āydukoḷḷabahudu)<br>Nim'ma tūkadallina badalāvaṇe (nīvu tūkavannu heccisiddīri/ nīvu tūkavannu kaḷedakoṇḍiddīri)<br>nim'ma carmada sukkugaṭṭavike<br>ettara kaḍimeyāgide<br>snāyugaḷu (mānsakhaṇḍagaḷu) kṣīṇisuttive.<br>Kūdalu uduruttide<br>kūdalu biḷiyāguttide.<br>Itare.
**15**	If yes to question 13, are you upset by this change?<br>Yes, very<br>Yes, somewhat<br>Yes, a little<br>No, not at all	Hadimūranē (13 nē) saṅkhyeya praśnege uttara"haudu" endādare, idarinda nīvu asamādhānagoṇḍiddīrā?<br>Haudu, tumbā<br>haudu, svalpamaṭṭige<br>haudu, svalpa<br>illa. Illavē illa	13Nē praśnege haudādare, ī badalāvaṇeyinda nīvu asamādhānagoṇḍiddīrā?<br>Haudu, tumbā<br>haudu, svalpamaṭṭige<br>haudu svalpa<br>illa, illavē illa	13 Nē praśnege uttara"haudu" endādare, ī badalāvaṇeyinda asamādhānagoṇḍiddīrā?<br>Haudu, tumbā<br>haudu, svalpamaṭṭige<br>haudu, svalpa<br>illa. Illavē illa	If your answer is yes for question 13 are you distressed with this change?<br>Yes, a lot<br>Yes, to some extent<br>Yes, a little<br>No, not at all	If your answer to the 13th question is 'yes', are you disappointed with the change?<br>Yes, to a great extent<br>Yes, to some extent<br>Yes, a little<br>No, Never	13 Nē praśnege uttara"haudu" endādare, nīvu ī badalāvaṇeyinda asamādhānagoṇḍiddīrā?<br>Haudu, tumbā<br>haudu, svalpamaṭṭige<br>haudu, svalpa<br>illa. Illavē illa	Asamādhān agoṇḍiddīrā	13 Nē praśnege uttara"haudu" endādare, nīvu ī badalāvaṇeyinda cintitarāgiddīrā?<br>Haudu, tumbā<br>haudu, svalpamaṭṭige<br>haudu, svalpa<br>illa. Illavē illa
**16**	Do you feel frail?<br>Very much so<br>A little<br>Not at all	Nīvu durbalarāgiddīri endu bhāvisuttīrā?<br>Tumbā anisuttide.<br>Svalpa<br>illavē illa	Nīvu durbalarāgiddīrā?<br>Khaṇḍitavāgi haudu<br>svalpa<br>illavē illa	Nīvu durbalarāgiddīri endu bhāvisuttīrā?<br>Khaṇḍitavāgi haudu<br>svalpa<br>illavē illa	Do you think you have become weak?<br>Yes, ofcourse<br>Yes, a little<br>No, not at all	Do you think that you have become weak?<br>Yes, Certainly<br>To some extent<br>No, never	Nīvu durbalarāgiddīri endu bhāvisuttīrā?<br>Khaṇḍitavāgi haudu<br>svalpa<br>illavē illa		Nīvu durbalarāgiddīri endu bhāvisuttīrā?<br>Khaṇḍitavāgi haudu<br>svalpa<br>illavē illa
**17**	Do you currently have difficulty in undertaking any of the following daily activities:	Nimage īga ī keḷagiruva yāva caṭuvaṭikegaḷannu māḍalu kaṣta anisuttade? Ondu mahaḍiya meṭṭilannu hattuvudu	Prastuta, ī keḷagina yāvudē dainandina caṭuvaṭikegaḷannu kaigoḷḷalu nimage tondare ideyē?	Nimage īga ī keḷagina yāva dainandina caṭuvaṭikegaḷannu māḍalu kaṣṭa anisuttade?	Which of the following activities are difficult for you?	Do you find it difficult to carry out any of the following activities?	Nimage īga ī keḷagina yāva dainandina caṭuvaṭikegaḷannu māḍalu kaṣṭa anisuttade?	Ettariruva	Nimage īga ī keḷagina yāva dainandina caṭuvaṭikegaḷannu māḍalu kaṣta anisuttade?<br>Ondu mahaḍiya meṭṭilannu hattuvudu

	Climbing a flight of<br>stairs?<br>Climbing several<br>flights of stairs?<br>Going up one or<br>several steps without<br>holding on to the<br>banister?<br>Squatting or kneeling?<br>Stooping or leaning<br>down to pick up<br>an object off the floor?<br>Getting up from the<br>floor without<br>holding on to<br>anything?<br>Getting out of a low<br>chair without<br>armrests?<br>Moving, generally,<br>from a sitting<br>position to a standing<br>position?<br>Carrying heavy objects<br>(large bags full<br>of shopping, saucepan<br>filled with<br>water, etc.)?<br>Opening a bottle or a<br>jar?<br>Using public<br>transport?<br>Getting in or out of a<br>car?<br>Doing your shopping?<br>Doing the housework<br>(making the<br>bed, hoovering, doing<br>the ironing,<br>washing the dishes,<br>etc.)?	ondakkinta heccu mahaḍiya<br>meṭṭilannu hattuvudu<br>byānisṭar(hyāṇḍ rēl) annu<br>hiḍidukoḷḷade ondu athavā<br>halavāru mahaḍiya<br>meṭṭilannu hattuvudu.<br>Bagguvudu athavā<br>maṇḍiyūruvudu<br>keḷage bidda vastuvannu<br>baggi tegeyuvudu<br>Yāvudē sahāyavillade<br>neladinda mēlēḷuvudu<br>kaḍime ettariruva ārms resṭ<br>illade iruva kurciyinda<br>mēlēḷuvudu<br>sāmān'yavāgi kuḷitallinda<br>mēlēḷuvudu<br>bhārada vastuvannu<br>ettuvudu(śāpiṅg tumbida<br>bhāravāda cīlagaḷu, nīru<br>tumbida bhārada pātre<br>ityādi)<br>bāṭali athavā jārina<br>muccaḷa tereyuvudu<br>Sārvajanika<br>vāhanagaḷannu baḷasuvudu<br>kārannu hattuvudu athavā<br>iḷiyuvudu<br>śāpiṅg māḍuvudu<br>mane kelasavannu<br>māḍuvudu (hāsigeyannu<br>hāsuvudu, maḍacuvudu,<br>vyākyūm klīn annu<br>māḍuvudu, baṭṭeyannu istri<br>māḍuvudu, pātre<br>toḷeyuvudu ityādi)	Meṭṭilugaḷannu<br>hattuvudu<br>tumbā meṭṭilugaḷannu<br>hattuvudu<br>kambagaḷannu<br>hiḍidukoḷḷade ondu<br>athavā halavāru<br>meṭṭilugaḷannu<br>hattuvudu<br>nelada mēle<br>kuḷitukoḷḷuvudu athavā<br>maṇḍiyūruvudu<br>Bāgi neladinda<br>vastuvannu<br>tegedukoḷḷuvudu<br>yāvudē sahāyavillade<br>neladinda mēlēḷuvudu<br>Kaigaḷillada saṇṇa<br>kurciyinda mēlēḷuvudu<br>sāmān'yavāgi<br>kuḷitukoḷḷuva<br>sthānadinda nintu<br>naḍeyuvudu<br>bhāravāda vastugaḷannu<br>oyyuvudu (vastugaḷinda<br>tumbida doḍḍa cīlagaḷu,<br>nīrininda tumbida<br>lōhada bōguṇi, ityādi)<br>Bāṭali athavā jār<br>tereyuvudu<br>sārvajanika sārigeyannu<br>baḷasuvudu<br>kārina oḷage hōguvudu<br>athavā horage baruvudu<br>vastugaḷannu kharīdi<br>māḍuvudu<br>manegelasavannu<br>māḍuvudu<br>(hāsigeyannu<br>sarimāḍuvudu, istri<br>māḍuvudu,<br>pātregaḷannu<br>toḷeyuvudu ityādi)	Ondu mahaḍiya meṭṭilannu<br>hattuvudu<br>ondakkinta heccu mahaḍiya<br>meṭṭilannu hattuvudu<br>byānisṭar(hyāṇḍ rēl) annu<br>hiḍidukoḷḷade ondu athavā<br>halavāru mahaḍiya<br>meṭṭilannu hattuvudu.<br>Maṇḍiyūruvudu<br>keḷage bidda vastuvannu<br>baggi tegeyuvudu<br>yāvudē sahāyavillade<br>neladinda mēlēḷuvudu<br>kaḍime ettariruva (kaigaḷu)<br>illade iruva kurciyinda<br>mēlēḷuvudu<br>sāmān'yavāgi kuḷitallinda<br>mēlēḷuvudu<br>bhāravāda vastuvannu<br>ettuvudu(vastugaḷinda<br>tumbida bhāravāda cīlagaḷu,<br>nīru tumbida bhārada pātre<br>ityādi)<br>bāṭali athavā jārina<br>muccaḷa tereyuvudu<br>Sārvajanika sārigeyannu<br>baḷasuvudu<br>kārannu hattuvudu athavā<br>iḷiyuvudu<br>śāpiṅg (vastugaḷannu<br>kharīdi) māḍuvudu<br>mane kelasavannu<br>māḍuvudu (hāsigeyannu<br>hāsuvudu mattu<br>maḍacuvudu, vyākyūm klīn<br>annu māḍuvudu, baṭṭeyannu<br>istri māḍuvudu, pātre<br>toḷeyuvudu ityādi)	Climbing up to<br>one floor<br>Climbing up<br>more than one<br>floor<br>Climbing several<br>floors without<br>holding railings<br>Kneeling down<br>Picking up the<br>fallen things<br>Getting up from<br>the floor without<br>anyone's help<br>Getting up from a<br>low armless chair<br>Usually getting<br>up from a seat<br>Lifting heavy<br>things (such as<br>heavy bags/<br>bucket of water<br>etc.)<br>Opening the lif of<br>a jar or bottle<br>Using public<br>transport<br>Getting into or<br>out of the car<br>Going for<br>shopping<br>Doing household<br>work (eg. making<br>bed, using<br>vaccum cleaner,<br>ironing, cleaning<br>utensils etc	Climbing the<br>steps<br>To the next<br>floor?<br>Climbing steps<br>to more than one<br>floor?<br>Climbing steps<br>to one or more<br>floors without<br>the support of<br>hand rail?<br>Kneeling down?<br>Picking things<br>that have fallen<br>down?<br>Getting up from<br>floor without<br>any support?<br>Getting up from<br>a chair without<br>arms?<br>Getting up<br>without support<br>from a sitting or<br>squatting<br>position?<br>Lifting any<br>heavy object<br>such as a bag<br>filled with<br>goods, a Water<br>pot etc.?<br>Removing the<br>cap/ thread of a<br>bottle?<br>Using a public<br>transport<br>vehicle/<br>boarding or<br>getting down<br>from bus etc.?<br>Boarding or<br>getting down<br>from a car?<br>Shopping<br>(buying goods)?	Ondu mahaḍiya<br>meṭṭilannu hattuvudu<br>ondakkinta heccu<br>mahaḍiya meṭṭilannu<br>hattuvudu<br>byānisṭar(hyāṇḍ rēl) annu<br>hiḍidukoḷḷade ondu athavā<br>halavāru mahaḍiya<br>meṭṭilannu hattuvudu.<br>Keḷage kuḷitukoḷḷuvudu/<br>maṇḍiyūruvudu<br>keḷage bidda vastuvannu<br>ettikoḷḷuvudu<br>yāvudē sahāyavillade<br>neladinda mēlēḷuvudu<br>Kaḍime ettariruva<br>kaigaḷillade (ārms resṭ)<br>iruva kurciyinda<br>mēlēḷuvudu<br>sāmān'yavāgi kuḷitallinda<br>mēlēḷuvudu<br>bhārada vastuvannu<br>ettuvudu(bhāravāda<br>cīlagaḷu, nīru tumbida<br>bhārada pātre ityādi)<br>bāṭali athavā jārina<br>muccaḷa tereyuvudu<br>sārvajanika sārigeyannu<br>baḷasuvudu<br>kārannu hattuvudu athavā<br>iḷiyuvudu<br>śāpiṅg (vastugaḷannu<br>kharīdi) māḍuvudu<br>Mane kelasavannu<br>māḍuvudu (hāsigeyannu<br>hāsuvudu mattu<br>maḍacuvudu, vyākyūm<br>klīn māḍuvudu,<br>baṭṭeyannu istri<br>māḍuvudu, pātre<br>toḷeyuvudu ityādi)		ondakkinta heccu mahaḍiya<br>meṭṭilannu hattuvudu<br>meṭṭilu hattuvāga gōḍe athavā<br>bēre yāvudara sahāyavillade<br>ondu athavā halavāru<br>meṭṭilugaḷannu hattuvudu<br>keḷage kuḷitukoḷḷuvudu/<br>maṇḍiyūruvudu<br>keḷage bidda vastuvannu<br>ettikoḷḷuvudu<br>Yāvudē sahāyavillade<br>neladinda mēlēḷuvudu<br>kaḍime ettara iruva kaigaḷillada<br>athavā hiḍiyalu ēnū illada<br>kurciyinda ēḷuvudu<br>sāmān'yavāgi kuḷitallinda<br>mēlēḷuvudu<br>bhārada vastuvannu ettuvudu<br>(bhāravāda cīlagaḷu, nīru<br>tumbida bhārada pātre ityādi)<br>bāṭali athavā jārina<br>muccaḷa tereyuvudu<br>Sārvajanika sārigeyannu<br>baḷasuvudu<br>kārannu hattuvudu athavā<br>iḷiyuvudu<br>śāpiṅg (vastugaḷannu kharīdi)<br>māḍuvudu<br>mane kelasavannu māḍuvudu<br>(hāsigeyannu hāsuvudu mattu<br>maḍacuvudu, guḍisuvudu,<br>oresuvudu, baṭṭeyannu istri<br>māḍuvudu, pātre toḷeyuvudu<br>ityādi)

						Doing household work (such as doing bed and Folding the bed, using vacuum cleaner Washing dishes etc.)?			
**18**	Does your muscle weakness limit your movement?<br>Yes, a lot<br>Yes, somewhat<br>Yes, a little<br>No, not at all	Nim'ma snāyu (mānsakhaṇḍada) daurbalyavu nim'ma calaneyannu mitagoḷisuttideyē?<br>Haudu, tumbā<br>haudu, svalpamaṭṭige<br>haudu, svalpa<br>illa, illavē illa	Nim'ma snāyu daurbalyavu nim'ma calaneyannu mitigoḷisuttadeyē?<br>Haudu, tumbā<br>haudu, svalpamaṭṭige<br>haudu svalpa<br>illa, illavē illa	Nim'ma snāyu (mānsakhaṇḍada) daurbalyavu nim'ma calaneyannu mitigoḷisuttadeyē?<br>Haudu, tumbā<br>haudu, svalpamaṭṭige<br>haudu, svalpa<br>illavē illa	Does you muscle weakness restrict you movement?<br>Yes, a lot<br>Yes, sometimes<br>Yes, a little<br>No, not at all	Do you think that the weakness of muscles has restricted your movement?<br>Yes, to a great extent<br>Yes, to some extent<br>Yes, a little<br>No, Never	Nim'ma snāyu (mānsakhaṇḍada) daurbalyavu nim'ma calaneyannu aḍḍigoḷisuttideyē?<br>Haudu, tumbā<br>haudu, svalpamaṭṭige<br>haudu, svalpa<br>illavē illa		Nim'ma snāyu (mānsakhaṇḍada) daurbalyavu nim'ma calaneyannu aḍḍigoḷisuttideyē?<br>Haudu, tumbā<br>haudu, svalpamaṭṭige<br>haudu, svalpa<br>illavē illa
**19**	If yes to question 18, for what reasons? (Choose as many answers as you like)<br>Fear of pain<br>Fear that you might not be able to<br>Fear of feeling tired after these activities<br>Fear of falling<br>Other:	Hadineṇṭaneya (18 nē) praśnege uttara"haudu" endādare, ēke? (Nimage iṣṭavādantaha ondakkinta heccina āykeyannu āydukoḷḷi)<br>Nōvina bhaya<br>nim'minda sādhyavāgadirabahudu emba bhaya<br>ī caṭuvaḷikegaḷa nantara daṇida bhāvaneya bhaya<br>bīḷuva bhaya<br>itare	18Nē praśnege haudādare, yāva kāraṇagaḷigāgi? (Nimage bēkāda uttaragaḷannu āykemāḍi)<br>Nōvina bhaya<br>nimage sādhyavāgadirabahudu emba bhaya<br>ī caṭuvaṭikegaḷa nantara daṇida bhāvaneya bhaya<br>bīḷuva bhaya<br>itare:	18 Nē praśnege uttara"haudu" endādare, ēke? (Nimage iṣṭavādantaha ondakkinta heccina āykeyannu āydukoḷḷabahudu)<br>Nōvina bhaya<br>nim'minda sādhyavāgadirabahudu emba bhaya<br>ī caṭuvaḷikegaḷa nantara daṇida bhāvaneya bhaya<br>bīḷuva bhaya<br>itare	If your answer is yes, Why? (Choose the relevant one or more option)<br>Scared of pain<br>Scared that you can't do it<br>Feeling of tiredness after the activity<br>Scared of falling<br>Other	If your answer to the 18th question is 'yes', why do you think so? (You can choose more than one of the following options)<br>Fear that you get pain<br>Fear that you get cannot complete the task<br>Apprehensive about the pain that you would get after doing the task<br>Fear that you triple down<br>Any other fear	18 Nē praśnege uttara"haudu" endādare, ēke? (Nim'ma āykegaḷannu āydukoḷḷabahudu)<br>Nōvina bhaya<br>nim'minda sādhyavāgadirabahudu emba bhaya<br>ī caṭuvaḷikegaḷa nantara daṇida bhāvaneya bhaya<br>bīḷuva bhaya<br>itare		18 Nē praśnege uttara"haudu" endādare, ēke? (Nim'ma āykegaḷannu āydukoḷḷabahudu)<br>Nōvina bhaya<br>nim'minda sādhyavāgadirabahudu emba bhaya<br>ī caṭuvaḷikegaḷa nantara daṇida bhāvaneya bhaya<br>bīḷuva bhaya<br>itare
**20**	Does your muscle weakness limit your sex life?<br>I am not sexually active<br>Yes, completely<br>Yes, somewhat	Nim'ma daihika durbalate nim'ma laiṅgika jīvanakke aḍḍiyāguttadeyē?<br>Nānu laiṅgika āsaktiyannu hondilla<br>haudu, sampūrṇavāgi<br>haudu, svalpamaṭṭige	Nim'ma snāyu daurbalyavu nim'ma laiṅgika jīvanavannu mitigoḷisuttadeyē?<br>Nānu laiṅgikavāgi sakriyavāgilla	Nim'ma snāyuvina durbalate nim'ma laiṅgika jīvanakke aḍḍiyāguttadeyē?<br>Nānu laiṅgika āsaktiyannu hondilla<br>haudu, sampūrṇavāgi<br>haudu, svalpamaṭṭige	Does your muscle weakness interrupt your married life?<br>Not interested<br>Yes completely<br>Yes sometimes	Is the weakness of muscles a hindrance to your sexual performance?<br>I don't have interest in sex	Nim'ma snāyuvina durbalate nim'ma laiṅgika jīvanakke aḍḍiyāguttadeyē?<br>Nānu laiṅgika āsaktiyannu hondilla (laiṅgikavāgi sakriyavāgilla)		Nim'ma snāyuvina (mānsakhaṇḍada) durbalate nim'ma laiṅgika jīvanakke aḍḍiyāguttadeyē?<br>Nānu laiṅgika āsaktiyannu hondilla (laiṅgikavāgi sakriyavāgilla)

	Yes, a little<br>No, not at all	haudu, svalpa<br>illa, illavē illa	haudu, sampūrṇavāgi<br><br>haudu, svalpamaṭṭige<br><br>haudu svalpa<br><br>illa, illavē illa	haudu, svalpa<br>illa, illavē illa	Yes a little<br>No, not at all	Yes, a total hindrance<br>Yes, to some extent<br>A little.<br>No, never	haudu, sampūrṇavāgi<br>haudu, svalpamaṭṭige<br>haudu, svalpa<br>illa, illavē illa		haudu, sampūrṇavāgi<br>haudu, svalpamaṭṭige<br>haudu, svalpa<br>illa, illavē illa
**21**	How has your participation in physical activities/sport changed?<br>Increased<br>Decreased<br>Unchanged<br>I have never participated in physical activities or sports	Nim'ma daihika caṭuvaṭike/ krīḍegaḷalli bhāgavahisuvike hēge badalāvaṇeyāgide?<br>Heccāgide<br>kaḍimeyāgide<br>ēnū badalāgilla<br>nānu endigū daihika caṭuvaṭike/ krīḍegaḷalli bhāgavahisilla	Daihika caṭuvaṭike/krīḍeyalli nim'ma bhāgavahisuvike badalāgideyē?<br>Heccāgide<br><br>kaḍimeyāgide<br><br>badalāvaṇe illa<br><br>nānu endigū daihika caṭuvaṭike athavā krīḍegaḷalli bhāgavahisalilla	Nim'ma daihika caṭuvaṭike/ krīḍegaḷalli bhāgavahisuvike hēge badalāvaṇeyāgide.?<br>Heccāgide<br>kaḍimeyāgide<br>ēnū badalāgilla<br>nānu endigū daihika caṭuvaṭike/ krīḍegaḷalli bhāgavahisilla	How do you think your physical activity and sports activities have changed?<br>It has increased<br>It has reduced<br>No change<br>I was not taking part in any physical/sport activity	How have your movements/ Physical activities and participation in sports changed?<br>It has increased<br>It has decreased<br>No Changes<br>I haven't participated in physical exercises or sports.	Nim'ma daihika caṭuvaṭike/ krīḍegaḷalli bhāgavahisuvike hēge badalāgide?<br>Heccāgide<br>kaḍimeyāgide<br>ēnū badalāgilla<br>nānu endigū daihika caṭuvaṭike/ krīḍegaḷalli bhāgavahisilla		Nim'ma daihika caṭuvaṭike/ krīḍegaḷalli bhāgavahisuvike hēge badalāgide?<br>Heccāgide<br>kaḍimeyāgide<br>ēnū badalāgilla<br>nānu endigū daihika caṭuvaṭike/ krīḍegaḷalli bhāgavahisilla
**22**	How has your participation in leisure activities (going out to eat, gardening, doing DIY, shooting/fishing, senior citizens clubs, playing bridge, going for a walk, etc.) changed?<br>Increased<br>Decreased<br>Unchanged<br>I have never participated in leisure activities	Biḍuvina (virāma) samayada caṭuvaṭikegaḷalli nim'ma bhāgavahisuvike hēge (tinnalu horage hōguvudu, tōṭagārike, nimage iṣṭavāda yāvudē kelasavannu māḍuvudu, śūṭiṅg/ mīnugārike, hiriya nāgarikara klabge hōguvudu, kārḍ(isṭīṭ) āḍuvudu, vākiṅg māḍuvudu) badalāgideyē?<br>Heccāgide<br>kaḍimeyāgide<br>ēnū badalāgilla<br>nānu endigū daihika caṭuvaṭike/ krīḍegaḷalli bhāgavahisalla.	Virāmada caṭuvaṭikegaḷalli (tinnalu hōguvudu, tōṭagārike, śūṭiṅg/mīnugārike, hiriya nāgarikara klab gaḷu, āḍuvudu, naḍeyuvudu, ityādi) nim'ma bhāgavahisuvike hēge badalāgide?<br>Heccāgide<br>kaḍimeyāgide<br>badalāvaṇe illa<br>nānu endigū virāmada caṭuvaṭikegaḷalli bhāgavahisalilla	Hēge nim'ma biḍuvina (virāmada) samayada caṭuvaṭikegaḷa bhāgavahisuvike (tinnalu horage hōguvudu, tōṭagārike, nimage iṣṭavāda yāvudē kelasavannu māḍuvudu, śūṭiṅg/ mīnu hiḍiyuvudu, hiriya nāgarikara klabge hōguvudu, kārḍ(isṭīṭ) āḍuvudu, vākiṅg māḍuvudu ityādi) badalāgide?<br>Heccāgide<br>kaḍimeyāgide<br>ēnū badalāgilla<br>nānu endigū virāmada caṭuvaṭikegaḷalli bhāgavahisalla.	Does your free time activities (going to eat, gardening, doing your favourite activity, shooting, fishing, going to senior citizen club, playing cards, walking etc.) have changed?<br>It has increased<br>It has become less<br>There has been no change<br>I have never engaged in any free time activities	How have your leisurely, outdoor activities changed? (For example, going out to eat, farming, doing an activity that you like, shooting/ fishing, going to a club, playing cards playing games and walking etc.)<br>It has increased<br>It has decreased<br>No Changes<br>I haven't participated in any leisurely activities	Nim'ma biḍuvina (virāmada) samayada caṭuvaṭikegaḷalli (tinnalu horage hōguvudu, tōṭagārike, nimage iṣṭavāda yāvudē kelasavannu māḍuvudu, śūṭiṅg/ mīnu hiḍiyuvudu, hiriya nāgarikara klabge hōguvudu, kārḍ(isṭīṭ) āḍuvudu, vākiṅg māḍuvudu ityādi) nim'ma bhāgavahisuvike hēge badalāgide?<br>Heccāgide<br>kaḍimeyāgide<br>ēnū badalāgilla<br>nānu endigū virāmada caṭuvaṭikegaḷalli bhāgavahisalla.		Nim'ma biḍuvina (virāmada) samayada caṭuvaṭikegaḷalli (samārambhagaḷalli bhāgavahisuvudu, tōṭagārike, nimage iṣṭavāda yāvudē kelasavannu māḍuvudu, makkaḷondige āṭa āḍuvudu, hiriya nāgarikara klabge hōguvudu, kārḍ(isṭīṭ) āḍuvudu, vākiṅg māḍuvudu ityādi) nim'ma bhāgavahisuvike hēge badalāgide?<br>Heccāgide<br>kaḍimeyāgide<br>ēnū badalāgilla<br>nānu endigū virāmada caṭuvaṭikegaḷalli bhāgavahisalla.
